# The *Drosophila* MAPK p38c Regulates Oxidative Stress and Lipid Homeostasis in the Intestine

**DOI:** 10.1371/journal.pgen.1004659

**Published:** 2014-09-25

**Authors:** Sveta Chakrabarti, Mickaël Poidevin, Bruno Lemaitre

**Affiliations:** 1 Global Health Institute, Station 19, EPFL, Lausanne, Switzerland; 2 Centre de Génétique Moléculaire (CGM), CNRS, Gif-sur-Yvette, France; The University of Texas Health Science Center at Houston, United States of America

## Abstract

The p38 mitogen-activated protein (MAP) kinase signaling cassette has been implicated in stress and immunity in evolutionarily diverse species. In response to a wide variety of physical, chemical and biological stresses p38 kinases phosphorylate various substrates, transcription factors of the ATF family and other protein kinases, regulating cellular adaptation to stress. The *Drosophila* genome encodes three p38 kinases named p38a, p38b and p38c. In this study, we have analyzed the role of p38c in the *Drosophila* intestine. The *p38c* gene is expressed in the midgut and upregulated upon intestinal infection. We showed that *p38c* mutant flies are more resistant to infection with the lethal pathogen *Pseudomonas entomophila* but are more susceptible to the non-pathogenic bacterium *Erwinia carotovora 15*. This phenotype was linked to a lower production of Reactive Oxygen Species (ROS) in the gut of *p38c* mutants, whereby the transcription of the ROS-producing enzyme *Duox* is reduced in *p38c* mutant flies. Our genetic analysis shows that p38c functions in a pathway with Mekk1 and Mkk3 to induce the phosphorylation of Atf-2, a transcription factor that controls *Duox* expression. Interestingly, *p38c* deficient flies accumulate lipids in the intestine while expressing higher levels of antimicrobial peptide and metabolic genes. The role of p38c in lipid metabolism is mediated by the Atf3 transcription factor. This observation suggests that p38c and Atf3 function in a common pathway in the intestine to regulate lipid metabolism and immune homeostasis. Collectively, our study demonstrates that p38c plays a central role in the intestine of *Drosophila*. It also reveals that many roles initially attributed to *p38a* are in fact mediated by *p38c*.

## Author Summary

The p38 mitogen-activated protein (MAP) kinase is a signaling pathway that is involved in both stress and immunity in various species from yeast to human. p38 kinases regulate transcription factors of the ATF family and other protein kinases that then induce cellular adaptation to stress to a wide variety of physical, chemical and biological stresses. The *Drosophila* genome encodes three p38 kinases named p38a, p38b and p38c. In this study, we have analyzed the role of p38c in the *Drosophila* intestine. The *p38c* gene is expressed in the digestive tract and up-regulated upon intestinal infection. We observed a lower production of Reactive Oxygen Species (ROS) in the gut of *p38c* mutants upon bacterial infection. Consistent with this observation, the transcription of the *Duox*, a gene encoding an enzyme that produces ROS, is reduced in *p38c* mutant flies. Our analysis shows that p38c induces the phosphorylation of Atf-2, a transcription factor that controls *Duox* expression. Interestingly, our study also shows that p38c and Atf3 function in a common pathway in the intestine to regulate lipid metabolism and immune homeostasis. Collectively, our study demonstrates that p38c plays a central role in the intestine of *Drosophila*.

## Introduction

In addition to its central role in digestion and absorption, the intestine serves as an interactive barrier against a wide variety of pathogens and commensals. This is especially true for insects such as *Drosophila*, which feed on rotting fruits and continuously ingest microbes. In recent years, *D. melanogaster* has emerged as a powerful model to investigate intestinal homeostasis and immunity [1]–[4].

Recent studies have shown that the *Drosophila* gut defense against bacterial infection involves (i) the production of ROS through the NADPH oxidase Duox, (ii) the production of antibacterial peptides through the Imd pathway, and (iii) the maintenance of gut homeostasis through regulation of stem cell activity [2]. Oral ingestion of bacteria induces the rapid synthesis of microbicidal ROS in the *Drosophila* gut by Duox [3]. The activity of Duox is triggered by the Gαq-phospholipase C-ß-Ca^2+^ pathway, which is itself initiated upon binding of an uncharacterized G-protein coupled receptor to uracil, a microbial ligand released from pathogenic bacteria [5], [6]. Duox is also regulated at the transcriptional level by the transcription factor Atf-2, downstream of a p38a-Mkk3-Mekk1-PGRP-LC (peptidoglycan recognition protein LC) pathway [7]. In addition to this ROS response, several antimicrobial peptides (e.g., Diptericin, Attacin) are produced in the gut under the control of the NF-kB protein Relish downstream of the Imd pathway [8]–[10]. This local immune response is triggered by the recognition of peptidoglycan from Gram-negative bacteria by the pattern recognition receptors PGRP-LC and PGRP-LE [11], [12]. Infection can also lead to intestinal damage, induced either by bacterial toxins or by the excessive production of ROS [13]–[17]. Stress response programs and increased epithelial renewal can then be deployed to repair the intestinal epithelium and maintain the integrity of the gut barrier. Epithelium renewal of the *Drosophila* gut is stimulated by the release of secreted ligands of the Unpaired and EGF families, which activate respectively the JAK/STAT and EGFR pathways in stem-cell like progenitor cells to promote their division and differentiation, thereby establishing compensatory homeostatic regulatory loops [2], [18].

We have recently described how an entomopathogenic bacterium, *Pseudomonas entomophila*, disrupts gut homeostasis in *Drosophila*. Although *P. entomophila* ingestion by *D. melanogaster* stimulates the transcription of genes encoding antimicrobial peptides (e.g. *Diptericin*) and epithelium renewal inducers (e.g. *upd3*), neither immune response nor epithelium renewal is observed. This is due to a general inhibition of translation in the intestine that affects all newly synthesized transcripts [16]. As a consequence, *D. melanogaster* succumb to *P. entomophila* infection because they are unable to repair the gut damage ensuing infection. This reduction of translation is a consequence of cellular damage to the intestine, caused by both host-derived ROS and the direct action of a pore-forming toxin produced by the pathogen. Thus the *P. entomophila* infection model provides a good system to probe the complex cross-talks between stress, repair and immune pathways during microbial infection [19].

To further study the interaction between stress response and host defense, we have now analyzed the role of the conserved p38 MAPK pathway in the *Drosophila* gut response. The p38 MAPK family has been involved in stress and immunity in both mammals and *Drosophila* [20]. In response to a wide range of physical, chemical and biological stresses, p38 kinases phosphorylate various substrates, such as transcription factors of the Activating Transcription Factor (ATF) family and other protein kinases, so as to regulate cellular adaptation to stress [21]. The *Drosophila* genome encodes for three p38 kinases named p38a, p38b and p38c. Mutations in *p38a* or *p38b* lead to an increased susceptibility to environmental stresses such as osmotic stress, heat stress or intestinal infection [22]–[24]. In contrast, *p38c* has been shown to regulate *dopadecarboxylase*, which encodes an enzyme involved in the formation of melanin in the epidermis following septic injury. This points to a possible role of p38c in wound healing [25].

In this study, we have analyzed the contribution of *Drosophila* p38c to the intestinal response to bacteria. We showed that *p38c* mutant flies are more resistant to *P. entomophila*, but are more susceptible to the non-pathogenic bacterium *Erwinia carotovora carotovora 15* (*Ecc15*). This phenotype is linked to a lower production of ROS in *p38c* mutants. We observed that the transcription of the ROS producing enzyme *Duox* is reduced in *p38c* mutant flies. In addition, we showed that Mekk1, Mkk3, p38c and Atf-2 function in a common pathway to control *Duox* transcription following infection. Finally, we observed that this pathway also regulates lipid homeostasis and basal antimicrobial peptide gene expression in an Atf3 dependent manner. Thus, our study delineates a pathway comprising Mekk1, Mkk3, p38c and Atf-2 that regulate the oxidative stress, immune response and lipid homeostasis in the intestine of *Drosophila*.

## Results

### *p38c* expression is up-regulated in the gut upon oral bacterial infection

**Figure 1:**
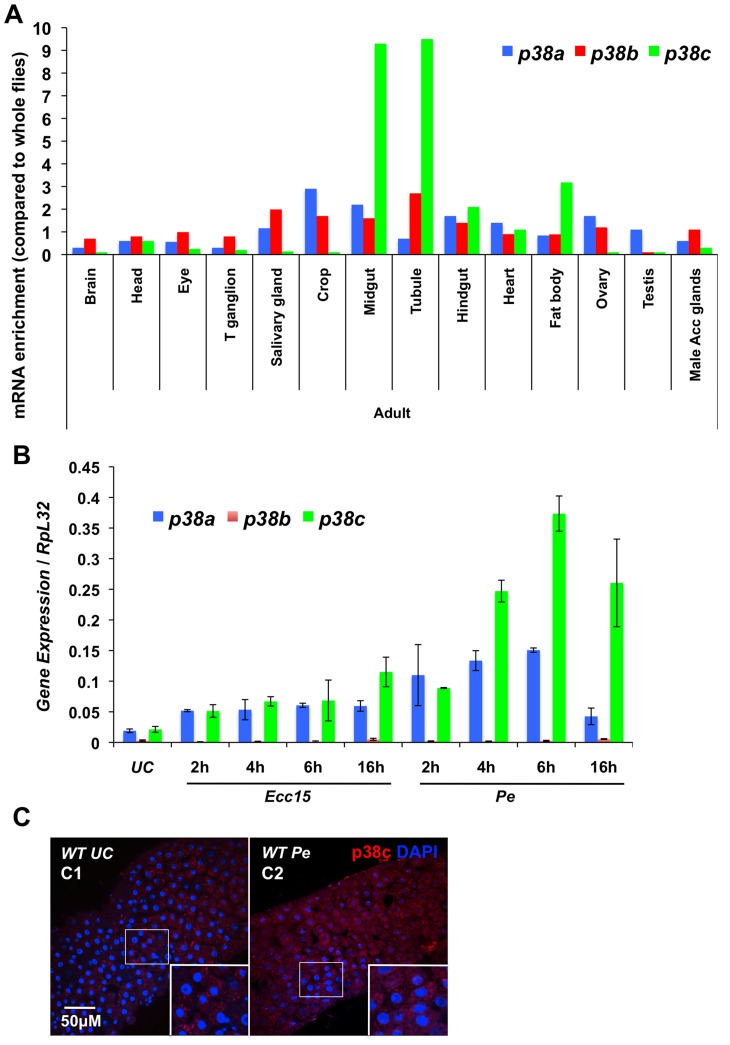
*p38c* is induced in the intestine following oral bacterial infection. (**A**) Data from Flyatlas showed an enrichment of p38c in the midgut [30]. Expression is shown as a ratio of mRNA enrichment for each gene in each tissue to the average mRNA enrichment for all the tissues. T ganglion: thoracic abdominal ganglion (**B**) *p38c* expression is the most induced *p38* gene in the midgut upon oral bacterial infection. The induction of the *p38* genes of *Drosophila* was monitored on gut RNA extracts of wild-type flies using RT-qPCR. Guts were collected at different time points (2, 4, 6 and 16 h) following oral infection with *Ecc15* and *P. entomophila (Pe)*. The level of induction of both *p38a* and *p38c* was higher following *P. entomophila* infection and peaked at 6 h post-infection. (**C**) Confoncal images of the anterior midgut stained with an anti-p38c serum of female flies either unchallenged (**C1**) collected 16 h after *P. entomophila* infection (**C2**). Insets show higher magnification. p38c is shown in red, nuclei are in blue. UC: unchallenged control. Diffuse or punctate signals corresponding to p38c protein were observed in the cytoplasm of enterocyte.

In *Drosophila*, three p38-MAPK-encoding genes, *p38a* (initially described as *mpk2*), *p38b* and *p38c*, have been identified (Figure S1A; [25]–[27]). Recent studies have revealed that p38a and p38b contribute to stress and immune responses in the *Drosophila* digestive tract [28], [29]. To date, the function of p38c in the intestine has not been described. Microarray data from FlyAtlas [30] showed that *p38c* transcripts are enriched in the midgut, Malpighian tubules and fat body of both larvae and adults when compared to *p38a* and *p38b* (Figure 1A). To characterize further the immune role of these MAPK genes, we monitored by RT-qPCR their expression in the intestine of flies orally infected with two Gram-negative bacteria, *Ecc15* and *P. entomophila*. This analysis revealed that *p38c*, and to a lesser extent *p38a*, is induced following infection with *Ecc15* and *P. entomophila* (Figure 1B). These results are consistent with previous microarray datasets analyzing gene expression profile in the gut of flies orally infected with *Ecc15* or *P. entomophila* (Figure S1B; [15], [16]). The enrichment of *p38c* in the gut and its high induction upon infection prompted us to investigate the role of this MAPK in the intestine. We first analyzed p38c localization in unchallenged and *P. entomophila*-infected intestine using a newly generated anti-p38c antibody, which was validated by an absence of signal in the *p38c* null mutant (*p38c^7B1^* [25]) (Figure S1C and S1D). p38c protein was localized to the cytoplasm of enterocytes (identified by their large nuclei) under unchallenged condition. Following *P. entomophila* oral infection the intensity of the p38c signals modestly increased (Figure 1C).

It has been suggested that the p38c kinase cannot be activated by phosphorylation due to a mutation that converts the TGY dual-phosphorylation site to TDH (Figure S2A). To test whether p38c is capable of kinase activity, we expressed and purified both GST- and Histidine- fusion derivatives of p38c in bacteria and carried out an *in vitro* kinase assay using the non-radioactive Kinase-Glo (Promega) kit with a mammalian GST-ATF2 fusion protein as an exogenous substrate (Figure S2B). We observed that GST-ATF2 was phosphorylated by both GST- and His-p38c fusion proteins. In addition, this kinase activity decreased in the presence of SB203580, a p38 inhibitor, which reduces its catalytic activity by binding to the ATP-binding pocket (Figure S2C). Altogether, our study shows that *p38c* is expressed in the gut, up-regulated upon infection and that the protein can function as a kinase at least *in vitro*, which suggests an important function of this MAPK in this organ.

### *p38c* mutants show a higher susceptibility to oral bacterial infection and to H_2_O_2_

**Figure 2:**
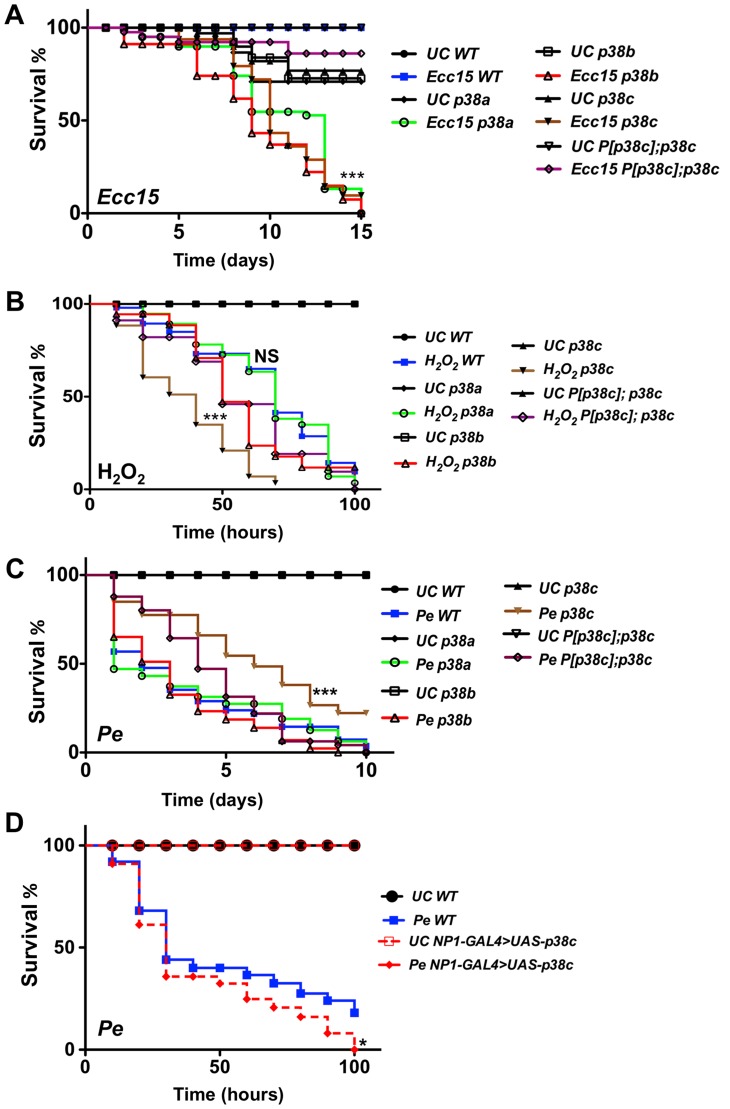
*p38c* flies were more resistance to *P. entomophila* infection. (**A**) A survival analysis of flies orally infected with the bacterium *Ecc15* reveals an increased susceptibility of *p38a^13^*, *p38b^156A^* or *p38c^7B1^* flies. ***: p<0.001 for *p38a^13^*, *p38b^156A^* or *p38c^7B1^* flies infected with *Ecc15*. (**B**) The *p38c^7B1^* mutant shows an increase susceptibility to 1% H_2_O_2_ as compared to the wild-type or the *p38a^13^* and the *p38b^156A^* mutants. ***: p<0.001 for *p38c^7B1^* mutant fed H_2_O_2_ and, NS: non-significant (p = 0.1223). (**C**) The *p38c^7B1^* mutant exhibits an increase resistance to oral infection with *P. entomophila*. ***: p<0.001 for *p38c^7B1^* mutant infected with *P. entomophila*. (**D**) Flies over-expressing *p38c* in the gut (genotype: *NP1-GAL4; UAS-p38c*) showed a slightly increased susceptibility to *P. entomophila* infection. WT: *NP1-GAL4; +* Of note, the intestines of *P. entomophila* infected flies over-expressing *p38c* displayed melanization in the most distal part of the midgut at the boundary with the hindgut. *: p<0.05 for (*NP1-Gal4>UAS-p38c*). UC: unchallenged in (**A**, **B**, **C**, **D**).

Previous studies have shown that the synthesis of antibacterial peptides under the control of the Imd pathway and production of ROS by Duox provide two complementary inducible defense mechanisms in the gut [5], [7], [10], [30]. The enrichment of *p38c* transcripts in this tissue and its induction post-infection pointed to a specific role of this MAPK in intestinal immune responses. We therefore analyzed the role of p38c in the resistance to oral infection with the non-lethal bacterium *Ecc15*. Figure 2A shows that *p38c^7B1^* mutant flies are more susceptible to *Ecc15* infection than wild-type flies. The level of susceptibility of *p38c^7B1^* flies is similar to that observed for *p38a* or *p38b* mutant flies (Figure 2A). To confirm that the higher susceptibility of *p38c^7B1^* flies is not due to the genetic background, we generated a fly line carrying both the *p38c^7B1^* mutation and a rescue transgene containing the *p38c* locus including 300 bp of upstream sequences (referred to as *P[p38c]*). *P[p38c];p38c^7B1^* flies showed a better survival to *Ecc15* infection compared to *p38c^7B1^* flies (Figure 2A). RT-qPCR analysis showed that *p38c* susceptibility is not due to an effect of *p38c* on the expression of *p38a* or *p38b* (Figure S3A).

We then investigated whether p38c affects the Imd pathway by measuring the expression of two antibacterial peptide genes, *Diptericin* and *Attacin-A*, in the gut of *p38c* deficient flies upon infection with *Ecc15*. We did not detect any effect of the *p38c* mutation on the expression levels of *Diptericin* after oral infection with this bacterium (Figure S3A). On the other hand, the basal levels of *Diptericin* and *Attacin-A*, as well as the induced levels of *Attacin-A* were higher in the intestines of *p38c* mutant flies (Figure S3A and B), but not in the rescued *P[p38c];p38c* flies. Thus the high susceptibility of *p38c^7B1^* flies to oral infection with *Ecc15* cannot be attributed to a lower activation of the Imd pathway.

As infection induces a ROS burst, we next investigated a possible link between p38c and resistance to oxidative stress. Previous studies have reported that p38a is required to resist oxidative stress [22]. However, the *p38a* strain used in these experiments (*p38a^1^* also called *mpk2*) was later shown to carry a deletion affecting *p38a* and its neighboring gene *p38c* (Figure S1A) [28]. To re-evaluate the contribution of each p38 member to oxidative stress resistance, flies carrying null mutations for either *p38a*, *p38b* or *p38c* were fed on a diet containing 1% H_2_O_2_. Figure 2B shows that *p38c^7B1^* but not *p38a^13^*, *p38b^156A^* or *P[p38c]*;*p38c* flies, are more susceptible than wild-type to 1% H_2_O_2_. This indicates that p38c (and not p38a as initially suggested) contributes to resistance to H_2_O_2_. Of note, survival of *p38c* over-expressing flies to 1% H_2_O_2_ was not significantly different compared to wild-type (Figure S3C). Collectively, our data show that all three *p38* genes contribute to survival to oral bacterial infection. It also highlights an important role of *p38c* in oxidative stress resistance.

### *p38c* is required for *P. entomophila*-induced translation inhibition

As opposed to *Ecc15*, *P. entomophila* is highly pathogenic to flies when fed at high doses. *P. entomophila* pathogenicity has been linked to its capacity to induce severe intestinal damage [15], [16]. We investigated the role of p38c in the defense against *P. entomophila* oral infection. Surprisingly, we observed that *p38c^7B1^* mutant flies are more resistant to infection with *P. entomophila* than either wild-type, *p38a^13^*, *p38b^156A^* mutants or *P[p38c]; p38c^7B1^* flies (Figure 2C and S4B). In this experiment, the survival of *P[p38c]; p38c^7B1^* was not statistically different from the wild-type. Nevertheless, the rescue effect was not observed at early time points possibly due to a lower level of *p38c* expression in *P[p38c]; p38c^7B1^* flies compared to wild-type (Figure S1C). We also analyzed the survival of the *p38c* over-expressing flies to *P. entomophila* infection and observed that the *p38c* over-expressing flies died slightly faster than the wild-type (Figure 2D).

**Figure 3:**
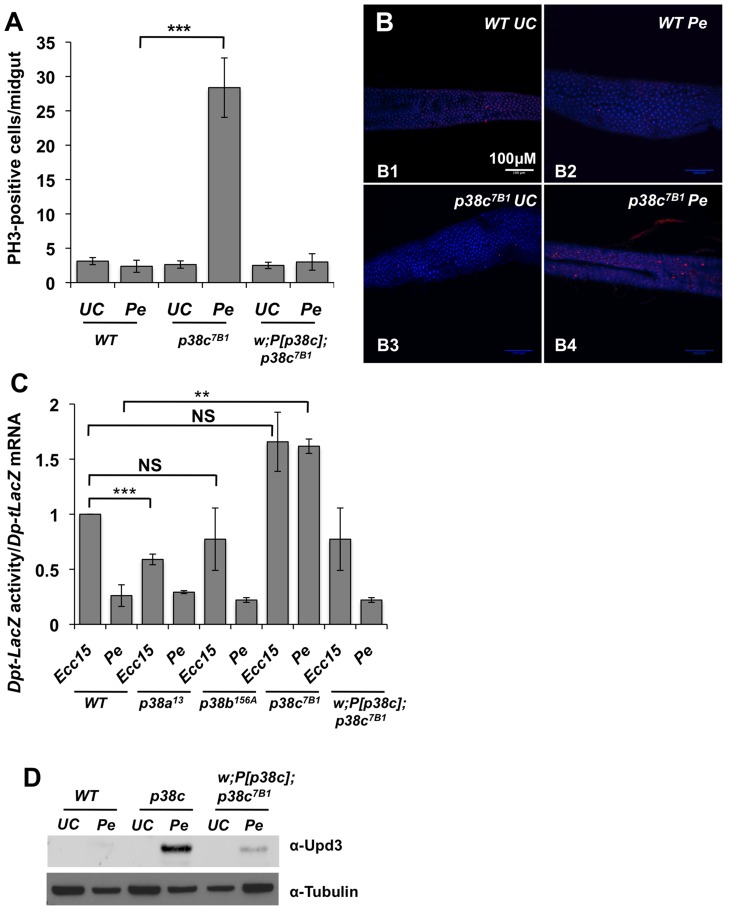
p*38c* contributes to *P. entomophila* mediated inhibition of translation. (**A**) *p38c^7B1^* flies showed an increased rate of epithelium renewal upon *P. entomophila* infection compared to wild-type flies. Stem cell division along the midgut was quantified using an anti-PH3-antibody. p<0.001 = *** (**B**) Sections of guts from *p38c^7B1^* flies orally infected with *P. entomophila* had higher number of dividing cells than their wild-type counterparts. Guts from unchallenged flies (**B1** and **B3**) or flies sampled 8 h after *Pe* ingestion (**B2** and **B4**) are shown. Small red cells (ISCs) immunostained using anti-PH3-antibody correspond to mitotic cells. DAPI: blue. (**C**) The Dpt-lacZ activity/*Dpt-lacZ* ratio (reflection the level of translation) in *P. entomophila* infected guts was higher in *p38c^7B1^* flies compared to *p38a^13^*, *p38b^156A^* or wild-type flies. This ratio was also higher upon *Ecc15* infection because infection with this pathogen also reduces translation, although at a lower level than that observed with *P. entomophila* [16]. Mean values of at least three experiments (N = 10 to 20 guts each) ± SE are shown. ** p<0.01, determined by Student's *t* test, NS: non-significant. The ratio Dpt-lacZ activity/*Dpt-lacZ* ratio observed upon *Ecc15* infection was higher but not significantly different in *p38c^7B1^* flies compared to wild-type. (**D**) Western blot analysis with an anti-Upd3 antibody revealed an increase Upd3 protein level in the guts of *p38c^7B1^* flies at 16 hr after *P. entomophila*.

Infection with high doses of *P. entomophila* leads to a rupture of gut integrity caused by the loss of stem cell activity and hence an absence of epithelium renewal [15], [16]. To decipher how p38c influences susceptibility to *P. entomophila* infection, we monitored the stem cell division rate in wild-type, *p38c^7B1^* and *P[p38c]; p38c^7B1^* flies upon *P. entomophila* infection. Epithelium renewal can easily be monitored by counting the number of mitotic stem cells along the midgut using an anti-phospho-histone 3 antibody. Figures 3A and 3B show that there was a higher mitotic index in the infected guts of *p38c^7B1^* mutants compared to wild-type or *P[p38c]; p38c^7B1^* flies. The *p38c^7B1^* mutant show a low level of stem cell activity under basal conditions and an overall gut structure similar to wild-type flies (Figure S4A), hence the increased intestinal stem cell activity upon infection is unlikely due to a defective gut organization but rather reflect a better capacity to repair the gut.

The lower epithelium renewal rate in *P. entomophila* infected guts is caused by a general inhibition of translation that impairs both immune and repair gene programs [16]. Translation inhibition can be monitored in the gut with a *Dpt-lacZ* transgene by analyzing the ratio between *Dpt-lacZ* ß-galactosidase activity and *Dpt-lacZ* transcripts, which reflect the extent of *Dpt-lacZ* translation and transcription respectively. A decrease in the ratio between ß-galactosidase activity and *Dpt-lacZ* transcript levels is indicative of a translation inhibition. As expected, the ratio of Lac-Z activity/*Lac-Z* mRNA was low in the gut of *P. entomophila* infected flies as compared to *Ecc15* infected flies (Figure 3C). This assay revealed an increased level of translation in *P. entomophila* infected gut of *p38c* flies (Figure 3C). In contrast, both *p38a^13^* and *p38b^156A^* flies exhibited a severe reduction in Dpt-lacZ translation, similar to the wild-type (Figure 3C). After infection or damage, epithelium renewal is stimulated by the release of a secreted ligand, Upd3, from stressed enterocytes, which activates the JAK/STAT pathway in progenitor cells to stimulate their division and their differentiation, thus establishing a homeostatic regulatory loop [14], [15]. Previous studies have shown that as a consequence of inhibition of translation, Upd3 was not produced in *P. entomophila* infected guts despite the strong induction of the upd3 gene. Figure 3D shows that, following *P. entomophila* infection, the level of Upd3 is higher in the intestines of the *p38c* mutants than in wild-type or *P[p38c];p38* flies. These results indicate that p38c participates in *P. entomophila*-induced translation inhibition in the gut. Thus, increased level of translation in *p38c^7B1^* flies could explain why *p38c^7B1^* flies survive better than wild-type.

### *p38c* regulates *Duox* transcription after infection

**Figure 4:**
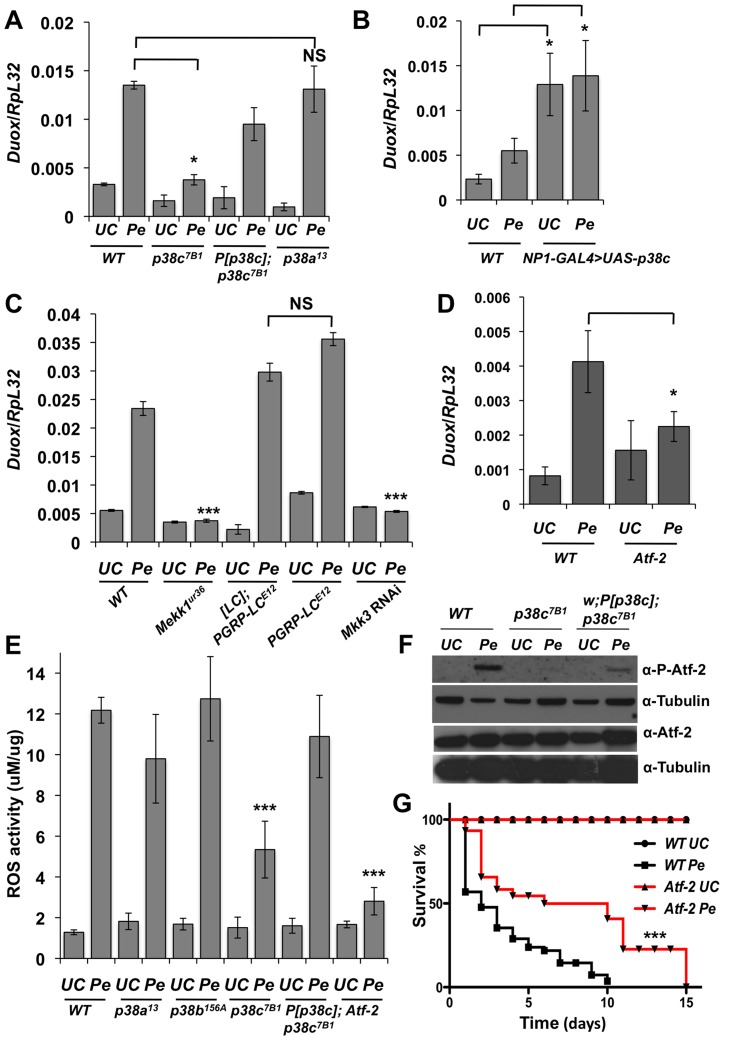
A Mekk1-Mkk3-P38c-Atf-2 pathway regulate *Duox* expression. (**A**) The induction of *Duox* upon *P. entomophila* infection is reduced in *p38c^7B1^* mutant flies. (**B**) Over-expressing *p38c* in the gut induced a higher level of *Duox* expression in the absence of infection. WT: *NP1-GAL4; +*. (**C**) Mekk1 and Mkk3 but not PGRP-LC regulates expression of *Duox* upon infection. Genotype: *w^1118^ (wild-type)*, *MEKK1^Ur36^*, *PGRP-LC^E12^*, *[PGRP-LC]; PGRP-LC^E12^* (a wild-type line with the same genetic background as *PGRP-LC^E12^*), and *NP1-GAL4;UAS-Mkk3IR*. (**D**) *Duox* expression was reduced in *Atf-2* flies upon infection. In (**A**, **C** and **D**) *Duox* expression was monitored by RT-qPCR performed with total RNA extracts from guts collected 2 h after *P. entomophila* infection. (**E**) Quantification of bacterial-induced ROS (H_2_O_2_) generation in gut extracts from adult female flies collected at 45 minutes post-infection with *P. entomophila* using Amplex Red reagent (Invitrogen). See methods for details. Mean values of three experiments (N = 10 guts each) ± SE are shown (**F**) Atf-2 is phosphorylated in wild-type but not in *p38c^7B1^* flies. The level of phosphorylation was only partially rescue in *p38c^7B1^, P[p38c]*. Western blot were performed on gut collected 4 h following oral infection with *P. entomophila*. The total levels of Atf-2 remain unchanged in all genotypes with or without infection. (**G**) *atf-2* deficient flies shows that an increase survival rate compared to wild-type orally infected with *P. entomophila*. Mean values of at least three experiments (N = 10 to 20 flies each) ± SE are shown. * p<0.05;, and NS: non-significant as determined by Student's *t* test. Kaplan-Meier log-rank test was used in (**G**) to determine statistical significance *** p<0.001.

*P. entomophila*-mediated translation inhibition is largely a consequence of the ROS produced by the Duox enzyme. Indeed, knocking-down *Duox* alleviates the inhibition of translation induced by *P. entomophila* [16] and increase short-term survival to *P. entomophila* (Figure S4B). A study has shown that *Duox* gene expression is regulated by the transcription factor Atf-2 downstream of p38a-Mekk3-Mekk1-PGRP-LC pathway [7]. However, the *p38a^1^* mutant used to analyze Duox regulation was the one that also contains a deletion affecting its neighboring gene *p38c* [28]. This raises the possibility that *Duox* is regulated by *p38c* and not by *p38a* as initially proposed. To test this hypothesis, we monitored *Duox* transcriptional activation in *p38a^13^* and *p38c^7B1^* single mutants. Figure 4A shows that *Duox* expression upon *P. entomophila* infection is lower in *p38c* compared to wild-type or to *p38a^13^* mutant flies. The genomic *P[p38c]* element rescues the loss of *Duox* induction in *p38c* mutants. Moreover, Figure 4B shows that overexpression of *p38c* is sufficient to induce *Duox* in the absence of infection. An increase in *Duox* gene expression should lead to higher levels of ROS, which are known to cause damage and to stimulate an epithelium renewal [15]. Consistent with this notion, a higher number of mitotic stem cells and a higher amount of Upd3 was found in flies that over-express *p38c* in absence of infection (Figures S4C–E). Using null mutations (*Mekk1^Ur36^, PGRP-LC^E12^*) and an RNAi construct (*Mkk3*), we then tested the effect of *PGRP-LC*, *Mkk3* and *Mekk1* mutations on the transcriptional induction of *Duox* by *P. entomophila*. Contrary to Ha *et al* (2009), we did not observe any effect of *PGRP-LC* on *Duox* expression but confirmed the requirement of both *Mkk3* and *Mekk1* (Figure 4C). Hence, p38c, Mkk3 and Mekk1, but not PGRP-LC or p38a, control the up-regulation of *Duox* in response to intestinal infection. This observation raises the hypothesis that the increased resistance of the *p38c^7B1^* mutants to *P. entomophila* infection is due to a reduced production of ROS by *Duox*. To test this hypothesis, we compared ROS levels in *p38c^7B1^* and wild-type fly guts upon *P. entomophila* infection using the Amplex Red reagent (Invitrogen). The data showed that ROS levels are indeed lower in the intestines of *p38c^7B1^* mutant flies than in wild-type (Figure 4E). Collectively, our study shows that *Duox* induction upon *P. entomophila* infection depends on p38c rather than p38a. The lower level of Duox-mediated ROS activity in *p38c* flies provides an explanation why translation is not inhibited in these flies and consequently why they survive better to *P. entomophila* infection.

### *p38c* and Atf-2 function in a same pathway to control *Duox* transcription following infection

A previous study combining an *in vivo* RNAi approach and promoter analysis has provided compelling evidence that *Duox* is directly regulated by the transcription factor Atf-2 [7]. We used a recently described fly line deleted for *atf-2* [31] to confirm that Atf-2 is indeed required for *Duox* transcription upon *P. entomophila* infection (Figure 4D). We observed that *atf-2* mutant flies exhibit lower ROS levels in the intestine following *P. entomophila* ingestion (Figure 4E) and are more resistant to this pathogen than wild-type (Figure 4G). Thus, the *atf-2* mutant phenocopies the *p38c^7B1^* mutants suggesting that p38c and Atf-2 may function in a common pathway to regulate *Duox* expression. In mammals, ATF2 is activated upon phosphorylation by p38 in response to various stresses [32]. Atf-2 is also phosphorylated following heat and osmotic stress in *Drosophila* S2 cells [31]. Figure 4F showed that Atf-2 is phosphorylated in the intestine in response to *P. entomophila* infection and that this phosphorylation is lost in *p38c* null flies. The genomic *P[p38c]* element partially rescues Atf-2 phosphorylation in *p38c* flies. As the total amount of Atf2 protein is not affected in *p38c* mutants, we conclude that p38c regulates Atf2 at the post-transcriptional level. Furthermore, the over-expression of p38c in the intestine is sufficient to phosphorylate Atf2 in the absence of infection (Figure S5A). This together with our *in vitro* analysis showing that p38c phosphorylates mammalian ATF2 (Figure S2B) strongly suggests that p38c directly phosphorylates Atf-2. Our results led us to conclude that a Mekk1/Mkk3/p38c/Atf-2 signaling pathway controls the expression of *Duox* expression upon intestinal infection.

### p38c and Atf3 function in a pathway regulating intestinal lipid homeostasis

**Figure 5:**
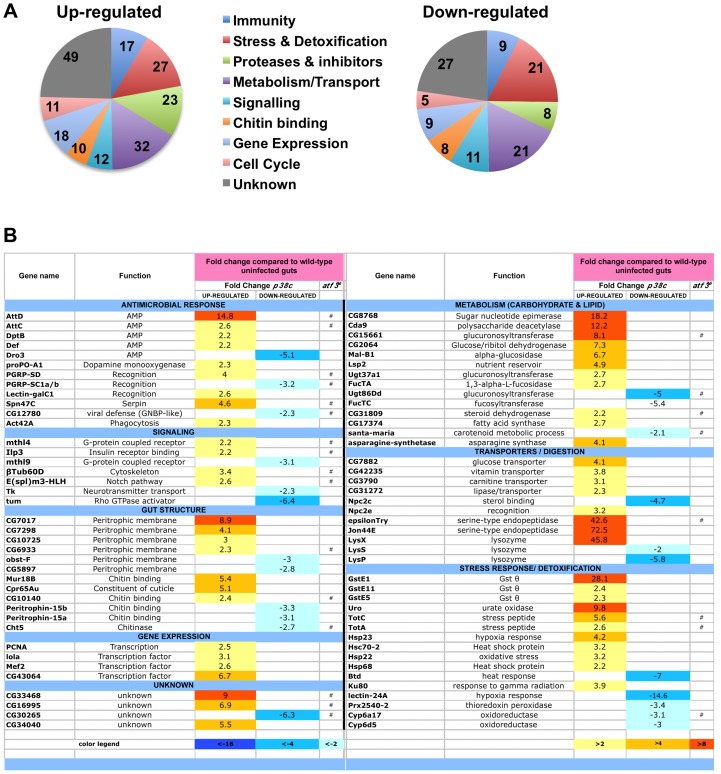
Genes involved in antimicrobial response, stress response, and metabolism are differentially regulated in *p38c^7B1^* flies. (**A**) Proportion of up-regulated (left) and down-regulated (right) genes in different Gene Ontology categories. (**B**) A selection of genes differentially regulated in *p38c^7B1^* fly guts (fold change compared to wild-type). Gene categories were determined by GO analysis on DAVID. Genes also affected in the *atf3^76^* mutant larvae as described in reference [33] are highlighted with an #. See Table S1 for complete list of genes.

**Figure 6:**
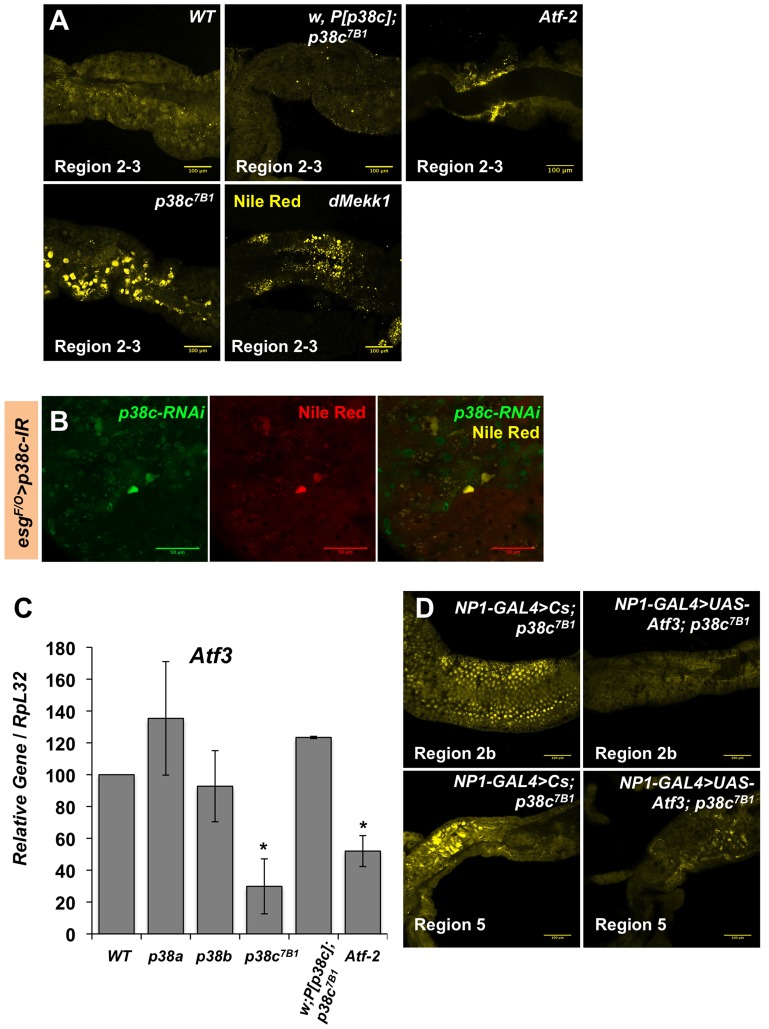
p38c and Atf3 function in a same pathway to control lipid homeostasis. (**A**) *p38c^7B1^* and *Mekk1^Ur36^* mutants accumulate lipids in regions of the midgut as observed by Nile Red. Enlarged lipid droplets were observed in the gut of *p38c* but not the wild-type or *p38c;P[p38c]* flies. *Atf-2* mutants showed a modest accumulation of neutral lipids as observed by Nile Red, when compared to the wild-type. (**B**) A lineage tracing system using *esg^ts^F/O* to silence *p38c* by RNAi in specific cells. Cells with reduced *p38c* expression (green) had increased lipid accumulation (yellow) relative to surrounding enterocytes. (**C**) RT-qPCR analysis of *Atf3* expression in 3–5 day old adult female fly intestines. Data are the mean of three repeats and error bars show standard error. * = p<0.05 determined by Student's *t* test. Genotypes are indicated on the x-axis, WT: *w^1118^*. (**D**) The over-expression of *atf3* in *p38c* mutant flies (genotype: *NP1-GAL4;UAS-ATF3,p38c^7B1^/p38c^7B1^*) restores a wild-type level of lipid in the gut.

We next explored whether p38c has additional roles in the intestine beyond regulating *Duox* expression. To gain insight into *p38c* function, we profiled genome expression in dissected intestines from unchallenged *w^1118^* and *w^1118^*, *p38c^7B1^* adult female flies using Affymetrix GeneChip *Drosophila* Genome 2.0 Arrays. Loss of *p38c* affected the expression of 408 transcripts, with 264 up-regulated and 144 down-regulated by at least 2-fold relative to the control. We used the GO clustering analysis tools and manual annotation to find functional categories within the 408 transcripts (Figure 5A–5B, see Table S1 for complete data set). As expected, one of the most represented GO category was the stress response with 48 genes modulated in this category. Consistent with the results described above, genes involved in oxido-reduction (e.g. oxidoreductases, GSTs) were differentially regulated in *p38c* intestine as compared to the wild-type. Our microarray analysis indicates that 26 immunity genes were up-regulated or down-regulated in *p38c* mutant flies. The *Drosophila* midgut is lined by a chitinous matrix, the peritrophic matrix (PM), which protects the midgut epithelium from abrasive food particles, digestive enzymes, and pathogen toxins [2]. 18 genes encoding for chitin-binding proteins were found to be modulated in the *p38c^7B^* mutant pointing to a role of this MAPK in the remodeling of the PM barrier (Figure 5B). The GO category with the largest number of genes was metabolism, notably sugar and lipid metabolism. Interestingly, many genes shown to be regulated in our analysis, notably genes involved in immunity, chitin and lipid metabolism (indicated with a # in Figure 5B) have previously been identified to be regulated by Atf3, a bZIP- transcription factor related to Atf-2 [33]. This finding and the observation that *p38c* expression is increased in *atf3^76^* mutant larvae [33] led us to explore a link between p38c and Atf3. One of the most striking phenotype of *atf3* mutant is an overload of lipids in the intestine of larvae [34]. Using an RNAi approach to knockdown Atf3 in the midgut of adult flies we also found a similar phenotype (Figure S6A). We also observed that *p38c* mutant flies accumulate lipids especially in two domains (R2b and R5) in the gut (Figure 6A for Nile-red in R2b and S6B for Oil-redO in whole intestine). To determine if the effect of the *p38c* knockdown on lipid metabolism is cell autonomous, we made positively marked clones of *p38c* RNAi knockdown using the *esg^ts^F/O* system [14], and examined lipid accumulation by Nile-red staining on whole guts. Whereas lipid accumulation was normal in cells outside the clones, there was accumulation of lipid in *p38c* knockdown clones (Figure 6B). Thus, the increased lipid accumulation in *p38c* mutant guts was not caused by altered feeding but to a specific requirement of p38c in the enterocytes. Of note, no lipid accumulation was observed in the intestine of *p38a* and *p38b* mutant flies (Figure S6C), whereas *Mekk1* flies showed a similar lipid accumulation in the intestine as *p38c* mutants (Figure 6A).

In mammals, the p38 pathway controls the transcription of the ATF3 gene in response to oxidative stress [34]. We thus investigated whether p38c regulates *Atf3* at the transcriptional level. Figure 6C shows that the *atf3* gene is expressed at a significantly lower level in unchallenged *p38c* mutant flies compared to wild-type. Conversely, over-expression of *p38c* in wild-type flies leads to an increased *atf3* expression (Figure S6D). Finally, we confirmed that *atf3* is epistatic to *p38c* as the over-expression of *Atf3* in the *p38c* mutant restores a wild-type level of lipids in the gut (Figure 6D). These data indicates that Mekk1, p38c and Atf3 function in a pathway required in the gut for lipid metabolism.

The results in the first part of this manuscript have shown that Mekk1 and p38c regulate the activity the transcription factor Atf-2 through its phosphorylation. Since p38c also regulates *Atf3* gene expression, we investigated whether the effect of p38c on *Atf3* transcription is mediated by Atf-2. Figure 6C shows that the amount of *Atf3* transcripts in the intestine is lower in *Atf-2* mutants than in wild-type, however the reduction is less marked than that observed in the *p38c* mutant background. Moreover, *Atf-2* deficient flies also accumulate lipids in the two gut regions but at a lower level than *Atf3* and *p38c* mutants (Figure 6A). Altogether, these results show that the effect of p38c on *Atf3* expression is partially mediated by Atf-2.

## Discussion

The p38 MAPKs have been implicated in the regulation of stress and immune responses in eukaryotes [21]. Despite several studies, the p38 MAPK pathway remains poorly characterized in *Drosophila*. In this study, we have analyzed the function of p38 MAPKs in the context of intestinal host defense and metabolic homeostasis, with an emphasis on p38c. Our study confirms a previous study indicating that both p38a and p38b are required to resist oral bacterial infection [28]. This function is not mediated through the Imd pathway, which regulates the antimicrobial response ([28], our data). Chen *et al* (2010) have proposed that p38a and p38b contribute to host defense by regulating Hsf1, a transcription factor which activates the expression of a large number of stress response genes, including many molecular chaperones (e.g. Heat Shock Proteins).

In this study, we have focused our attention on p38c, which has been somewhat neglected so far. The only function that had been previously attributed to p38c was the regulation of the *dopadecarboxylase* gene in the wounded epithelium [25]. Here, we show that the *p38c* gene is strongly expressed in the gut compared to *p38a* and *p38b* and is strongly up-regulated upon bacterial infection (Buchon 2009, this study). Like p38a and p38b, p38c is also required to survive an infection with the non-lethal bacterium *Ecc15*. Moreover, we show *in vitro* assay reveals that p38c has indeed a kinase activity and can phosphorylate mammalian ATF2. Although it remains to be shown how p38c gets activated following infection, our results suggest that it functions as a kinase rather than a scaffold protein. A role for p38c as a kinase is also supported by the observation that Atf-2 phosphorylation is impaired in *p38c* deficient flies.

A first surprising result was the observation that *p38c* flies survive better to oral infection with *P. entomophila*. As *P. entomophila* pathogenesis is caused by an excessive activation of stress pathways caused by ROS-induced damage, we investigated the link between Duox activation and p38c. Our study shows that *Duox* expression is regulated by p38c and not by p38a as previously reported [7]. Consistent with this, a lower level of ROS was observed in *p38c* flies infected with *P. entomophila*. The lower expression of *Duox* in *p38c* flies provides an explanation why these flies are susceptible to *Ecc15*, an infection model in which Duox contributes to survival [30], while being more resistant to *P. entomophila*, an infection model in which ROS production by Duox contributes to pathogenesis [16].

Consistent with Ha et al. 2009 we observed that *Duox* expression is regulated by the Atf-2 transcription factor upon phosphorylation by a MAPK pathway involving p38c, Mkk3 and Mekk1. However, we did not find PGRP-LC to be involved in *Duox* regulationin response to *P. entomophila* infection, indicating that this cascade is not triggered by peptidoglycan recognition as previously reported [7]. To date, the factors that trigger the p38c MAPK cascade following infection remains to be identified. One possibility is that uracil, a bacterial product shown to modulate Duox activity by the Gαq-Phospholipase Cß-Ca^2+^ pathway [6], is involved in the activation of the p38c MAPK pathway. Our study further shows that p38c (and not p38a as initially proposed) contributes to resistance to H_2_O_2_. This indicates that the p38c is involved both in the production of extracellular ROS by regulating *Duox* transcription and in the protection against the cytotoxic effect of ROS. Our identification of a role of p38c in the antioxidant response is an important step toward a better understanding of ROS metabolism in the intestine. This response may not involve Atf-2 because we did not observed an increased susceptibility of *atf-2* mutant flies to H_2_O_2_ or a phosphorylation of Atf-2 in wild-type flies in response to H_2_O_2_ (Figure S5C and D). It would be interesting to characterize how p38c mediates this antioxidant response. A recent paper has suggested that p38 could affect the activity of glutathione-S-transferases (GSTs) by modulating its substrate specificities in *Drosophila* [35]. In this line, our microarray shows that the expression of many genes involved in oxido-reduction (GSTs) and detoxification are modulated by p38c.

Previous study using an *in vivo* RNAi study has shown that reduction of *Atf-2* expression in the fat body results in reduced triglyceride storage and a decreased survival under starvation conditions [36]. Multiple genes that control triglyceride metabolism, including the *PEPCK* gene, which encodes a key enzyme required for triglyceride synthesis via glycerol-3-phosphate, are expressed at lower levels in *Atf-2* knockdown flies. During the course of our study, we noticed that *p38c* mutant flies are leaner than wild-type flies and have also reduced TAG levels (Figure S7A and S7B). Consistent with the decrease in TAG reserves, *p38c^7B1^* flies (but not *p38a^13^* and *p38b^156A^*) succumb more rapidly to acute starvation, in which flies are only given a source of water but no source of nutrition (Figure S7C). These observations suggest that p38c and Atf-2 also function in a common pathway to regulate lipid homeostasis in the fat body. Hence, it is likely that the regulation Atf2 transcription factor by p38c is not restricted to the intestine.

Another unexpected observation was that *p38c* deficient flies accumulate lipids in the intestine. Such accumulation is similar to the phenotype described for *Atf3* mutant larvae [33]. In addition, in both *atf3* and *p38c* deficient flies, genes involved in lipid metabolism and immunity are up-regulated in the intestine. This observation suggested that p38c and Atf3 function in a common pathway in the gut to regulate lipid metabolism and immune homeostasis. In agreement with this, we could show that *Atf3* is regulated in the intestine at the transcriptional level by p38c. This effect is partially mediated by Atf-2 as supported by a RT-qPCR analysis and the intermediate lipid accumulation phenotype of *Atf-2* mutants. Thus, *Atf3* gene expression would be regulated by p38c in the intestine by both Atf2-dependent and Atf2-independent pathways. Many immune genes are expressed at higher than wild-type levels in the intestine of *p38c* deficient flies in the absence of infection. The mechanism underlying the higher activation of these immune genes in *p38c* flies was not identified, however Rynes and colleagues have shown that immune gene activation in *atf3* larvae requires Relish, the Imd pathway transcription factor [33]. Nevertheless, the effect of p38c on antimicrobial peptide gene expression could be direct if Atf-2 or Atf3 directly regulated the transcription of these genes under basal conditions. Alternatively, a higher Imd pathway activity could be an indirect consequence of the rupture of gut homeostasis.

The present study allowed us to better delineate the p38 MAPK responsive pathway in *Drosophila*, revealing a central role for p38c in the gut. It also revealed that many roles initially attributed to p38a [22], notably in the resistance to H_2_O_2_ and to starvation, are in fact mediated by p38c. Seisenbacher et al. [29], have previously shown that p38a also contribute to the resistance to osmotic shock using a fly line deleted for both *p38a* and *p38c*. To clarify this point, we monitored the resistance to osmotic stress of single p38 mutants. Figure S8B shows that only the double mutant *p38a^1^ (Mpk2)* affecting both *p38a*, *p38c* but neither *p38a* nor *p38c* single mutant exhibit an increase susceptibility to high salt (Figure S8). Our study also confirms a previous study showing that p38b contributes to the resistance to osmotic shock ([29], Figure S8). To avoid any confusion in the future, we propose that the *p38a^1^* (also called *mpk2*) strain should be renamed *p38a-c^del^*.

There are substantial parallels between the regulation of ATF transcription factors and p38 in *Drosophila*, *C. elegans*, and mammals. This is probably a legacy of the ancestral role of this pathway in animal stress responses. In *C. elegans*, PMK1 (the p38 homolog) regulates the phosphorylation of ATF7 (the Atf-2 homologue) to control the transcription of immune genes in the intestine upon ingestion of pathogenic bacteria [37]. The regulation of ATF2 by p38-mediated phosphorylation [38], as well as interactions between ATF2, ATF3 and p38 have also been observed in mammals. ATF3 is up-regulated through the p38 pathway in response to oxidative or anisomycin stresses [39], [40]. Another study reports that ATF2 can activate the expression of ATF3 in response to stress in colonic cancer cells [41]. Moreover, ATF3 has also been shown to regulate lipid metabolism in pancreatic ß-cells of mice [42]. Our study suggests that ATF2 and ATF3 could play a fundamental role in the intestine of mammals. We believe that inter-specific comparisons of p38 MAPK pathway functions should allow us to better understand the mechanism underlying p38 activation and to decipher the specific roles that this pathway has acquired in different organs.

## Materials and Methods

### *Drosophila* stocks and rearing

*Canton^S^* (*Can^S^*) and *w^1118^* flies were used as wild-type controls. The following fly lines were used in this study: *y^1^ w^67c23^; p38a^13^* [28], *y^1^ w^67c23^; p38b^156A^* [28], *yw; p38b^d27^/CyO,y^+^* [23], *PGRP-LC^E12^* [43], *p38a^1^* [22], *MEKK1^Ur36^* [44], *p38c^7B1^* [25], *[PGRP-LC];PGRP-LC^E12^* [12], *Atf-2* (*PBac* [*32*] *Atf-2^c06467^*) [31], *Gαq^1^* [5], *UAS-Atf3* [45], w*; Df(3R)w6/TM6C (Bloomington stock 7251), *NP1-GAL4 (II)* (or *Myo1A-Gal4* [10], *UAS-Duox-IR* (GD2593, VDRC), *UAS-mkk3-IR* (KK108550, VDRC) and *UAS-Atf3-IR* (26741, Bloomington TRiP line). *esg-Flip-Out system* (*esgF/O*, [14]: *esg-Gal4, tub-Gal80^TS^; UAS-FLP act_FRT_CD2_FRT_Gal4, UAS-GFP* flies were crossed with *UAS-p38c-IR* (KK103439, VDRC).

For RNAi (IR) studies, F1 progeny carrying one copy of the driver as well as one copy of the UAS-IR were raised at 18°C during their larval and pupal development, and then moved to 29°C for 8 days to activate the UAS-IR. *Drosophila* stocks were maintained using standard fly medium comprising of 6% cornmeal, 6% yeast, 0.62% agar, 0.1% fruit juice, supplemented with 10.6 g/L moldex and 4.9 ml/L propionic acid. All stocks were maintained at 25°C on a 12 h light/12 h dark cycle unless otherwise stated.

### Plasmids and transgenic lines

*UAS-p38c* construct: A full-length cDNA of *p38c* was amplified from total cDNA of *Oregon^R^* flies and cloned into the pDONR207 Gateway vector (Invitrogen) and subsequently sub-cloned in the pTW (Drosophila Genomics Resource Center plasmid) transgenesis vector and used to generate transgenic flies. A fly line carrying the transgene on the third chromosome was established and used as *UAS-p38c*.

*P[p38c*] rescue construct: To generate a rescue transgene of *p38c*, we amplified by PCR a fragment comprising the coding region of p38c with the 3′UTR and 300 bp of the upstream sequence (corresponding to the sequence between *p38c* and *p38a*,). The amplicon was cloned into pCasper4 plasmid using the restriction sites Not1 and Xho1, and used for generating transgenic flies according to standard procedures. Fly line carrying the transgene on the second chromosome, and was introgressed into the *p38c^7B1^* mutant.

### Bacterial strains and infection experiments

*Erwinia carotovora carotovora 15 (Ecc15)* and *Pseudomonas entomophila (Pe)* are Gram-negative bacteria described in [9], [46]. Both bacteria were cultured overnight in LB medium at 29°C. For oral infection, batches of 20 adult female flies of 3 to 5 day old age were starved for 2 h at 29°C in an empty vial before being transferred to a fly vial with infection solution and maintained at 29°C. The infection solution consisted of an equal volume of 100× concentrated pellet from an overnight culture of *Ecc15* or *Pe* (OD_600_ = 200) with a solution of 5% sucrose (1∶1) was deposited on a filter disk that completely covered the surface of standard fly medium. Flies were incubated for one day at 29°C on the contaminated filter, after which they were transferred to fresh vials containing standard medium without living yeast.

### Oxidative stress, osmotic stress and starvation stress assay

For survivals to oxidative stress, flies were fed on a standard medium containing 1% H_2_O_2_ (Sigma). To assess sensitivity to osmotic stress, eggs were collected on apple agar plates and 20–30 eggs per genotype were transferred on to standard medium containing 0.2 M NaCl and survival to adulthood was recorded. Starvation experiments were performed with 2–3 day old female flies in batched of 20 flies. Flies were transferred to vial containing 0.62% agarose to prevent dessication of flies (supplemented with moldex and propionic acid). The survival analysis was done by counting dead flies every 10 h.

### RT-qPCR

Twenty dissected guts (crop, midgut and hindgut without Malpighian tubules) were collected in Trizol (Invitrogen) and total gut RNA was extracted according to manufacturer's instructions. Quality and quantity of RNA was determined using NanoDrop ND-1000 spectrophotometer. 1 µg of RNA was used to generate cDNA using SuperScript II (Invitrogen, Carlsbad, California, United States). RT-qPCR was performed using dsDNA dye SYBR Green I (Roche Diagnostics, Basel, Switzerland). Expression values were normalized to *RpL32*. Primers sequences used is provided in Table S2.

### Quantitative measurements of β-galactosidase activity

Ten to twenty female adult guts were dissected and homogenized in Z buffer [47] and centrifuged for 10 minutes at 10 000 r.p.m. (4°C). β-Galactosidase activity was measured as described in [48] and normalized to the protein concentration determined by Bradford assay (Sigma). Results are represented as nmol product formed/min/mg protein.

### Monitoring the level of translation

To determine the level of translation we monitored the ratio between Dpt-lacZ (ß-galactosidase) activity (as described above) and *Dpt-lacZ* transcript level (normalized on the amount of RpL32) in gut extracts of *Dpt-lacZ* flies. The ratio obtained from flies collected at 16 h post *Ecc15* infection was set up as 1. Reduction of this ratio indicates an inhibition of translation.

### Production of the antibody anti-p38c

The anti-p38c antibody was produced by immunizing rabbits with GST-p38c fusion protein following the 28-day protocol (Eurogentec, Belgium).

### Imaging and immunohistochemistry

For immunofluorescence, guts were dissected in 1X PBS, fixed for 20 minutes in PBS and 0.1% Tween 20 (PBT), and 4% paraformaldehyde; then stained with primary antibody [1/100 anti-p38c (this study); 1/500 anti-PH3 (Upstate/Millipore)] in PBT+2% BSA. Secondary staining was performed with Alexa594 anti-rabbit antibodies (Invitrogen). DNA was stained with 4′,6- diamidino-2-phenylindole DAPI (Sigma). The stained gut tissue was mounted in the antifading agent Citifluor AF1 (Citifluor Ltd.). PH3 positive cells were counted along the gut with Axioplot imager (Zeiss).

### Reactive oxygen species measurement

Ten adult female guts (including the crop) per genotype were dissected and collected in 300 µl Ringer's solution (pH 7.2) 45 minutes after oral infection with *P. entomophila*. Dissected guts were homogenized and centrifuged to remove debris. ROS level in the adult guts was monitored by the addition of 100 µl reaction buffer (50 µM Amplex Red, Invitrogen # A12222; 0.2 µM horseradish peroxidase from Sigma prepared in *Drosophila* Ringer's solution) to 20 µl homogenized gut extract. Fluorescence intensity was measured with a fluorescence microplate reader using excitation wavelength of 530 nm and emission wavelength of 590 nm. A standard curve was plotted with a range of H_2_O_2_ dilutions and used to determine the amount of H_2_O_2_ and normalized to the amount of protein.

### Western blot analysis

At least 30 female adult guts were dissected and homogenized in lysis buffer (0.5%NP40, 500 mM NaCl, 500 mM Tris-HCl pH 7.4, 20 mM EDTA, 10 mM NaF, 2 mM benzamidine, and a cocktail of protease and phosphatase inhibitors from Roche). Protein quantification was done with Bradford reagent (Sigma), before equal amount of protein was loaded and separated on a NUPAGE 4–12% Bis-Tris precast gel (Invitrogen) under reducing conditions. Following transfer onto a nitrocellulose membrane and blocking in 2% bovine serum albumin in PBT for 1 h, the membrane was incubated with the appropriate primary antibodies at 4°C overnight. Secondary antibodies were incubated for 2 h at room temperature and chemiluminescence signal was detected using ECL (GE Healthcare) according to the manufacturer's instructions. Primary antibodies used were at the following dilutions: α-tubulin antibody 1∶10000 (Sigma), α-Atf-2 1∶1000 and α-P-Atf-2 1∶1000 [31], α-Upd3 1∶1000 ([16]; gift from Yu-Chen Tsai) and α-p38c 1∶1000. Secondary antibodies used were: anti-rabbitHRP 1∶1000 (Dako); anti-mouseHRP 1∶1000 (GE Healthcare).

### Nile Red and Oil Red O staining

Three to four day old female fly guts were dissected and fixed in 500 µl of 4% paraformaldehyde for 20 minutes at room temperature. For nile Red staining (to stain intracellular lipid droplets), fixed guts were incubated in Nile Red solution (diluted 1∶100 in 1X PBS from a 100 µg/ml stock solution in acetone) [48] for 15 minutes at room temperature and then washed with 1X PBS three times before mounting on a slide in 70% Glycerol. The tissue was imaged with a Zeiss LSM700 upright confocal microscope under the 20X/0.8 NA objective. At least 10 guts per genotype were imaged per experiment.

For Oil Red O staining, fixed guts washed twice with MilliQ water, 100% propylene glycol and the incubated in propylene glycol for 10 minutes. The tissue was then incubated in 0.5% Oil Red O in propylene glycol at 60°c for 30 minutes. 10 ml of Oil Red O staining solution was prepared by mixing 6 ml (0.1% Oil Red O) in isopropanol and 4 mL MilliQ H_2_O, which was filtered through a 0.45 um syringe filter. After 30 minutes incubation in Oil Red O solution the gut tissue was washed twice in 85% propylene glycol at RT, thrice in MilliQ water before mounting onto a slide in 70% Glycerol. Samples were imaged with a Leica MZ16F dissecting microscope.

### Triacylgylceride assay

For colorimetric triacylglyceride assays, five 3–5 day old female flies were washed in phosphate-buffered saline (1X PBS) and then homogenized in 200 µl of PBST. The homogenate was heat inactivated at 70°C and then centrifuged (3,000 rpm; 5 minutes; 4°C). 20 µl aliquots of the supernatant were assessed in 96-well plates with the Triglyceride reagent and Free Glycerol Reagent (Sigma). Lipid levels were normalized to the protein contents.

### Promega's Kinase-Glo luminescent kinase assay

Kinase-Glo is a homogeneous non-radioactive method for determining the activity of purified kinases by quantifying the amount of ATP remaining in a solution following a kinase reaction. Luminescence signal correlated with the amount of ATP present and inversely proportional to the amount of kinase activity. To determine EC_50_ of P38c-his, a serial dilution of the kinase was added to the reaction buffer (40 mM Tris-HCl, 0.1 mg/ml BSA, 20 mM MgCl2 and 10 µM ATP) with 1 µg ATF2-GST in a final volume of 50 µl. After 30 minutes of reaction, 50 µl Kinase-Glo Reagent was added to each well and incubated for another 10 minutes at room temperature. The p38 kinase inhibitor SB203580 compound (Invitrogen) was dissolved in dimethyl sulfoxide (DMSO) and then added to the reaction buffer with the kinase prior to the addition of the substrate. For the 0 µM SB203580 condition, DMSO was added alone. Luminescence of each well was measured by TecanF200 microplate spectrofluorometer.

### Analysis of whole genome mRNA expression by Affymetrix Droso2.0 chips

The microarray analysis was performed on 3 independent biological repeats. RNA from 30 guts (crop, midgut and hindgut without Malpighian tubules) per genotype (*w^1118^* and *p38c^7B1^*) of 3 to 5 days old females from was isolated by TRIzol extraction, and purified with RNA clean up purification kits (Macherey Nagel). The quality of RNA was assessed on Agilent 2100 bioanalyzer chips. For each sample, 100 ng of total RNA was amplified and labeled using the GeneChip IVT Labeling Kit according to the manufacturer's protocol. Affymetrix Drosophila Genome 2.0 arrays were hybridized with 30 mg of labeled cRNA, washed, stained, and scanned as described in the Affymetrix Manual. Statistical analyses were performed using the R and Bioconductor statistical packages. All the genes integrated in the analysis shown in Figure 5 were differentially expressed by at least 2-fold with a p value <0.05. The raw data from the microarray experiment have been deposited in ArrayExpress (accession number E-MTAB-2740).

### Statistics

Each experiment was repeated independently a minimum of three times (unless otherwise indicated), error bars represent the standard error of the mean of replicate experiments (unless otherwise indicated). Statistical significance was determined using Student's t test or log–rank test on GraphPad Prism, and P values of <0.05 = *, <0.01 = ** and <0.001 = *** were considered significant.

## Supporting Information

Figure S1 *Drosophila* p38 deletions used in this study. (**A**) Schematic representations of the p38 mutations used in this study. The *p38a,p38c* double mutant (previously described as *p38a^1^* or *mpk2^1^*) is also shown. Figure is adapted from [22], [23], [28]. Deletions are marked with red-dashed lines. (**B**) Expression of *p38* genes upon infection with *Ecc15* and *P. entomophila* infection. Microarray data were extracted from [10], [16]. The fold change upon infection (compared to sucrose-fed flies) is shown 4 h and 16 h post-infection. (**C**) Use of an anti- p38c sera revealed that p38c is induced in wild-type and to a lesser extent in *P[p38c]; p38c^7B1^* rescue flies. The specificity of the p38c anti-sera was confirmed by the absence of signal in *p38c^7B1^* mutant flies. Western blot was performed on gut extracts collected 16 h following *P. entomophila* infection. (**D**) The p38c antibody was validated by lack of any staining in *p38c^7B1^* mutant flies (uninfected, **D1**; 16h after infection, **D2**). The p38c staining was restored in *p38c^7B1^* mutant flies containing a genomic rescue of *p38c* (uninfected, **D3**; 16h after infection, **D4**). p38c is shown in red, nuclei are in blue. UC: unchallenged control.
(TIF)

Figure S2 Analysis of *p38c* kinase activity. (**A**) An alignment of the amino acid sequences of p38c in *Drosophila* (Dp38c), and the human p38α. The conserved residues required for kinase activity (verified in mammalian studies) are indicated with a triangle. The orange filled triangles show the mutated residues of p38c that could lead to a loss/decrease of kinase activity (see UniProt accession Q16539 for details). An alignment of the kinase domain of the three *Drosophila* p38 genes with the human *p38α* (right bottom panel). The conserved phosphorylated motif is marked in red. A graphical representation of p38c with the MAPK domain marked in purple containing the TDH motif (left bottom panel). (**B**) A kinase titration curve using varying concentration of recombinant p38c-His protein amounts revealed an EC_50_ of 0.3 µM. (**C**) The compound SB203580 inhibits p38c-His kinase activity for the substrate GST-ATF2 protein, at µM range. The compound was added to the reaction buffer with the substrate before adding the kinase. Concentrations of SB 203580 used are indicated below.
(TIF)

Figure S3 The expression of antimicrobial peptide genes is increased in the *p38c* mutant gut. (**A**) RT-qPCR analysis of *Dpt*, *p38a* and *p38b* expression in intestines of adult females either unchallenged or collected at 16h after oral infection with *Ecc15* or *P. entomophila*. *Dpt* was up-regulated under basal conditions in the *p38c^7B1^* mutant flies. *** p<0.001, determined by Student's *t* test. Data are the mean of three repeats and ± SE are shown. (**B**) Up-regulation of *AttA* expression in the *p38c^7B1^* mutant was observed with or without infection. RT-qPCR was performed on total RNA extract from adult females intestine collected at 16 h after oral infection with *Ecc15*. UC: Unchallenged. NS: Not Significant (p = 0.3386); * p<0.05; *** p<0.001, determined by Student's *t* test. Data are the mean of three repeats and ± SE are shown. (**C**) Susceptibility to oxidative stress of wild-type flies (*NP1-GAL4>Cs*) and flies over-expressing *p38c* (*NP1-GAL4>UAS-p38c*) in the midgut fed on a diet with 1% H_2_O_2_. Despite an increase resistance at early time points, the survival of flies over-expressing *p38c* did not differ significantly from the wild-type based on a Kaplan-Meier log-rank.
(TIF)

Figure S4 Contribution of p38c and Atf-2 to *P. entomophila* pathogenicity. (**A**) Structure and general organization of the gut of *p38c* deficient flies is similar to the wild-type. Green: visceral muscles stained with phalloidin-Alexa488; blue: nuclei marked with DAPI. (**B**) *Gαq^1^* mutant, *Duox* RNAi and *p38c/Df(3R)w6* flies exhibited an increased resistance to oral infection with *P. entomophila*. UC: unchallenged, Kaplan-Meier log-rank test used to determine statistical significance compared to the wild-type *** p<0.001. (**C**) *P. entomophila* infected *atf-2* flies showed an increased mitotic index compared to wild-type flies. Flies over-expressing *p38c* (*NP1-GAL4; UAS-p38c*) had a higher mitotic index in absence of infection. Stem cell division along the midgut was quantified 8 h post-infection using an anti PH3-antibody. p<0.001 = *** (**D**) Immunostaining of guts revealed a higher number of mitotic stem cell in unchallenged flies over-expressing *p38c* (*NP1-Gal4>UAS-p38c*) D1. D2 flies were collected 8 h post-infection with *P. entomophila*. Mitotic stem cells: red; DAPI: blue. (**E**) Western blot analyses showed that flies over-expressing *p38c* have higher amount of Upd3 protein. Western blot was performed with protein extract of gut from flies either unchallenged or collected 16 h post-infection with *P. entomophila*. Flies that over-expressed p38c were subjected to *P. entomophila* mediated inhibition of translation and as consequence did not express Upd3 and did not show an increase of mitotic activity.
(TIF)

Figure S5 Atf-2 functions downstream of p38c in the regulation of Duox. (**A**) Western blot analysis showed an increase of Atf-2 phosphorylation where *p38c* was over-expressed. Guts were collected 4 h post-infection with *P. entomophila*. The total levels of Atf-2 remain unchanged in all genotypes with or without infection. (**B**) RT-qPCR analysis of *Duox* expression in various genetic backgrounds. Total RNA was extracted from guts of flies either unchallenged or collected 2 h after *P. entomophila* infection. *Duox* was highly expressed in absence of infection in flies over-expressing p38c but not in the *Atf-2* mutant background. The induction of *Duox* upon *P. entomophila* infection was reduced in ‘*p38a^1^*’ mutant flies (deficient for both *p38a* and *p38c*) that over-express a functional *p38a-GST* fusion confirming that p38c is required for *Duox* up-regulation. The precise genotypes were 1. WT: *NP1-GAL4/+*, 2. *NP1-Gal4/+;UAS-p38c/+*, 3. *Atf-2, NP1-Gal4/Atf-2, +; UAS-p38c/+* and 4. *NP1-GAL4/+; p38a^1^, UAS-p38a-GST/p38a^1^*,+. (**C**) *Atf3* and *Atf-2* mutant flies showed similar susceptibility to H_2_O_2_ as wild-type flies. A Kaplan-Meier log-rank test used to determine statistical significance. (**D**) Western blot analysis showed that Atf-2 phosphorylation was not induced when flies were fed on 1% H_2_O_2_. Flies were collected at 4 h post-feeding.
(TIF)

Figure S6 Increase accumulation of lipids in *p38c^7B1^* fly intestines. (**A**) Silencing *Atf3* by RNAi in the gut of adults leads to accumulation of lipids as observed by Nile Red staining. Different regions (Region 1, Regions2–3, Region 5) of the gut are shown for both the WT (*NP1-GAL4/+* top panels) and *Atf3* RNAi (*NP1-GAL4; UAS-ATF3-IR* bottom panels). (**B**) Oil-Red O stainings revealed a higher amount of lipids in the gut of *p38c^7B1^* flies compared to the wild-type and *P[p38c]; p38c^7B1^* flies. (**C**) *p38a^13^* and *p38b^156A^* flies showed wild-type amounts of lipid in the intestine (WT: *w^1118^*). (**D**) The expression of *atf3* increased in flies over-expressing *p38c* in the intestine WT: *NP1-GAL4; +* Data are the mean of three repeats and error bars show standard error. * p<0.05 as determined by Student's *t* test.
(TIF)

Figure S7 p38c flies have reduced TAG store. (**A**) *p38c^7B1^* adult female flies appeared leaner (slightly smaller) than their wild-type (*w^1118^*) counterparts. Flies were imaged 3 days post-eclosion. (**B**) Shown are the levels of TAG in *p38* and *Atf-2* mutants relative to wild-type flies. Flies were maintained on standard *Drosophila* medium (see methods) for 3–5 days prior to TAG analysis. TAG measurements were normalized for the total amount of protein (µg/mg of protein). This analysis revealed that *p38c* and *Atf-2* flies have lower levels of total TAG. Mean values of at least three experiments (N = decapitated 5 flies) (**C & D**) *p38c* mutant flies and to a lesser extent *atf-2* flies exhibited an increase susceptibility to a starvation stress as compared to wild-type flies. 3–5 day-old females (genotype indicated in the panel) were fed on 1% agar vials. Kaplan-Meier log-rank test was used to determine statistical significance ** p<0.01. (**E**) Expression of a *p38c-IR* element with the ubiquitous driver *Da-GAL4* reduced *p38c* expression without affecting *p38a* or *p38b*. RT-qPCR was performed on 3–5 days old flies. Genotype: *Da-GAL4/+* and *Da-GAL4/UAS-p38cIR*.
(TIF)

Figure S8 *p38a*, *p38c* double mutants are sensitive to salt stress. Embryos were placed on standard medium (**A**) or standard medium with 0.2 M NaCl (**B**), and the total numbers of offspring were counted. Panel B shows that *p38b^156A^* and *mpk2* (deficient for both *p38a* and *p38c*) flies show an increase susceptibility to osmotic stress. WT: *w^1118^* and other genotypes are indicated in the figure.
(TIF)

Table S1 List of up-regulated and down-regulated genes in the *Drosophila* gut of *p38c^7B1^* flies. Gene name, Probe ID, function, fold change and biological Gene ontology is indicated. Genes affected in the *atf3^76^* in reference [34] and *Rel^E20^* in reference [10] are marked with +. Values represent the fold change compared to wild-type guts.
(XLSX)

Table S2 List of qPCR primer sequences used in this study.
(DOCX)
