# Supplementary figures and images for: The Drosophila MAPK p38c Regulates Oxidative Stress and Lipid Homeostasis in the Intestine
Source: PLoS Genet. 2014 Sep 25;10(9):e1004659. doi: 10.1371/journal.pgen.1004659 (PMC4177744; doi:10.1371/journal.pgen.1004659)

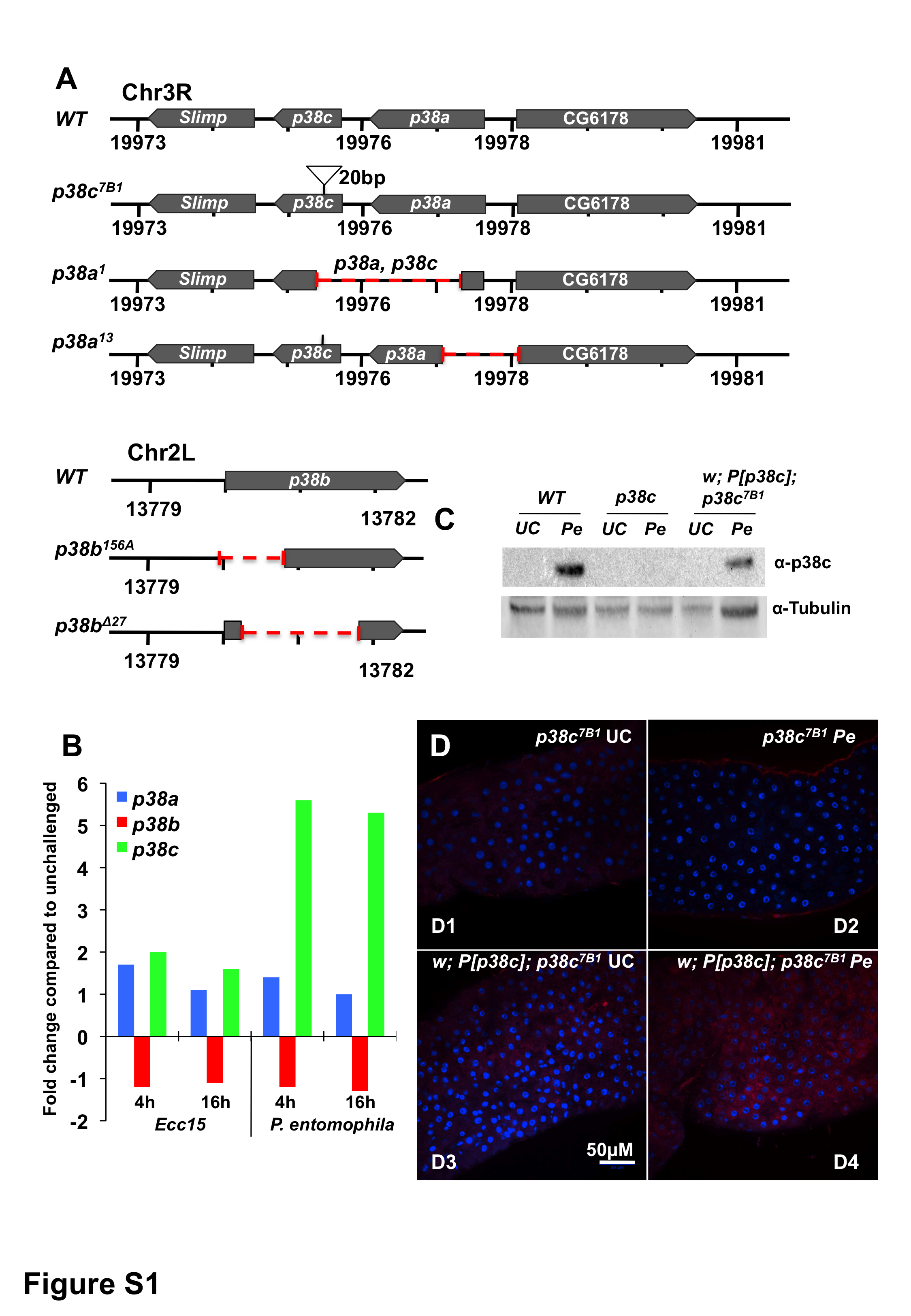

Supplement: Figure S1 — Drosophila p38 deletions used in this study. (A) Schematic representations of the p38 mutations used in this study. The p38a,p38c double mutant (previously described as p38a1 or mpk21) is also shown. Figure is adapted from [22], [23], [28]. Deletions are marked with red-dashed lines. (B) Expression of p38 genes upon infection with Ecc15 and P. entomophila infection. Microarray data were extracted from [10], [16]. The fold change upon infection (compared to sucrose-fed flies) is shown 4 h and 16 h post-infection. (C) Use of an anti- p38c sera revealed that p38c is induced in wild-type and to a lesser extent in P[p38c]; p38c7B1 rescue flies. The specificity of the p38c anti-sera was confirmed by the absence of signal in p38c7B1 mutant flies. Western blot was performed on gut extracts collected 16 h following P. entomophila infection. (D) The p38c antibody was validated by lack of any staining in p38c7B1 mutant flies (uninfected, D1; 16h after infection, D2). The p38c staining was restored in p38c7B1 mutant flies containing a genomic rescue of p38c (uninfected, D3; 16h after infection, D4). p38c is shown in red, nuclei are in blue. UC: unchallenged control. (TIF) [file pgen.1004659.s001.tif]

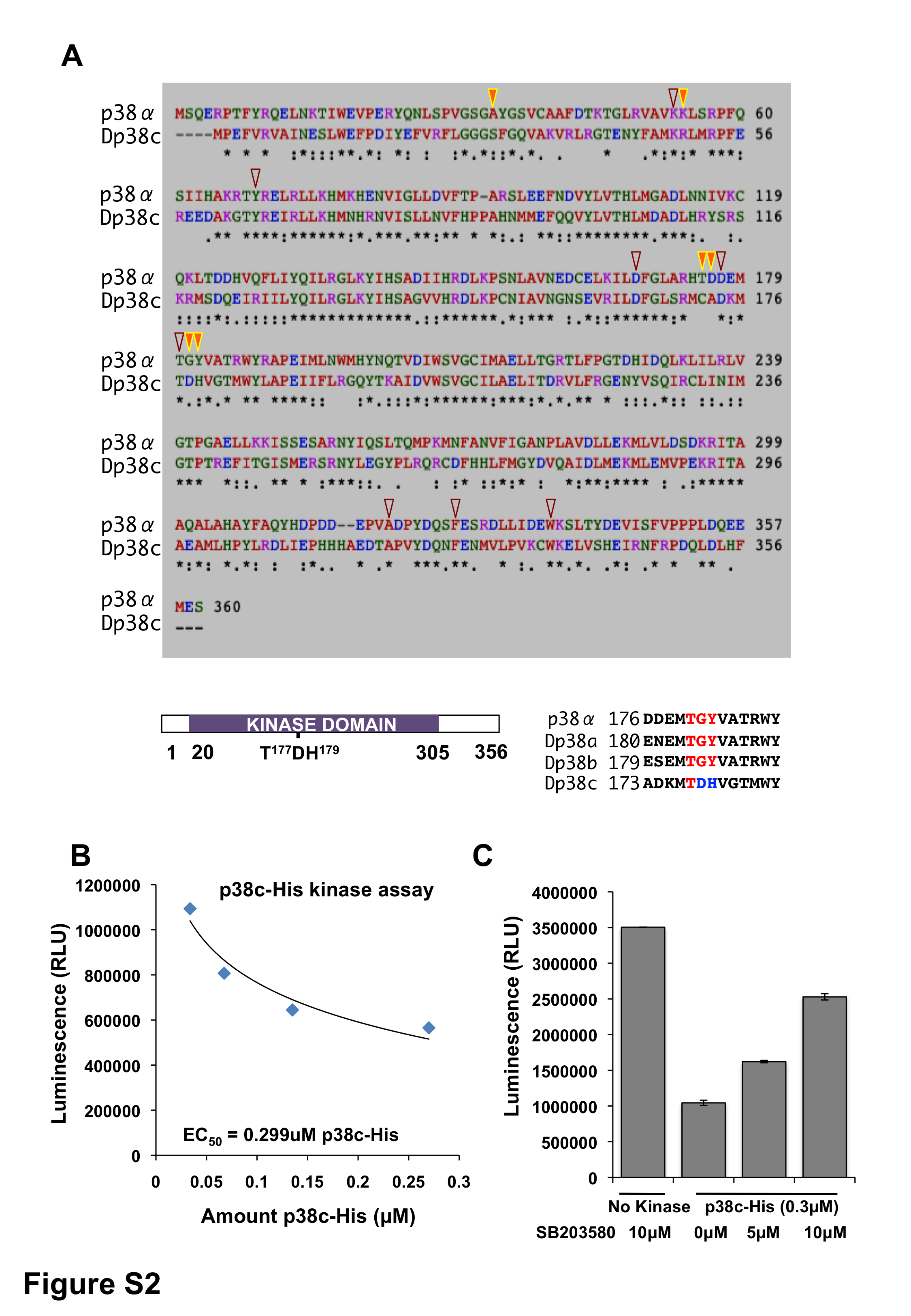

Supplement: Figure S2 — Analysis of p38c kinase activity. (A) An alignment of the amino acid sequences of p38c in Drosophila (Dp38c), and the human p38α. The conserved residues required for kinase activity (verified in mammalian studies) are indicated with a triangle. The orange filled triangles show the mutated residues of p38c that could lead to a loss/decrease of kinase activity (see UniProt accession Q16539 for details). An alignment of the kinase domain of the three Drosophila p38 genes with the human p38α (right bottom panel). The conserved phosphorylated motif is marked in red. A graphical representation of p38c with the MAPK domain marked in purple containing the TDH motif (left bottom panel). (B) A kinase titration curve using varying concentration of recombinant p38c-His protein amounts revealed an EC50 of 0.3 µM. (C) The compound SB203580 inhibits p38c-His kinase activity for the substrate GST-ATF2 protein, at µM range. The compound was added to the reaction buffer with the substrate before adding the kinase. Concentrations of SB 203580 used are indicated below. (TIF) [file pgen.1004659.s002.tif]

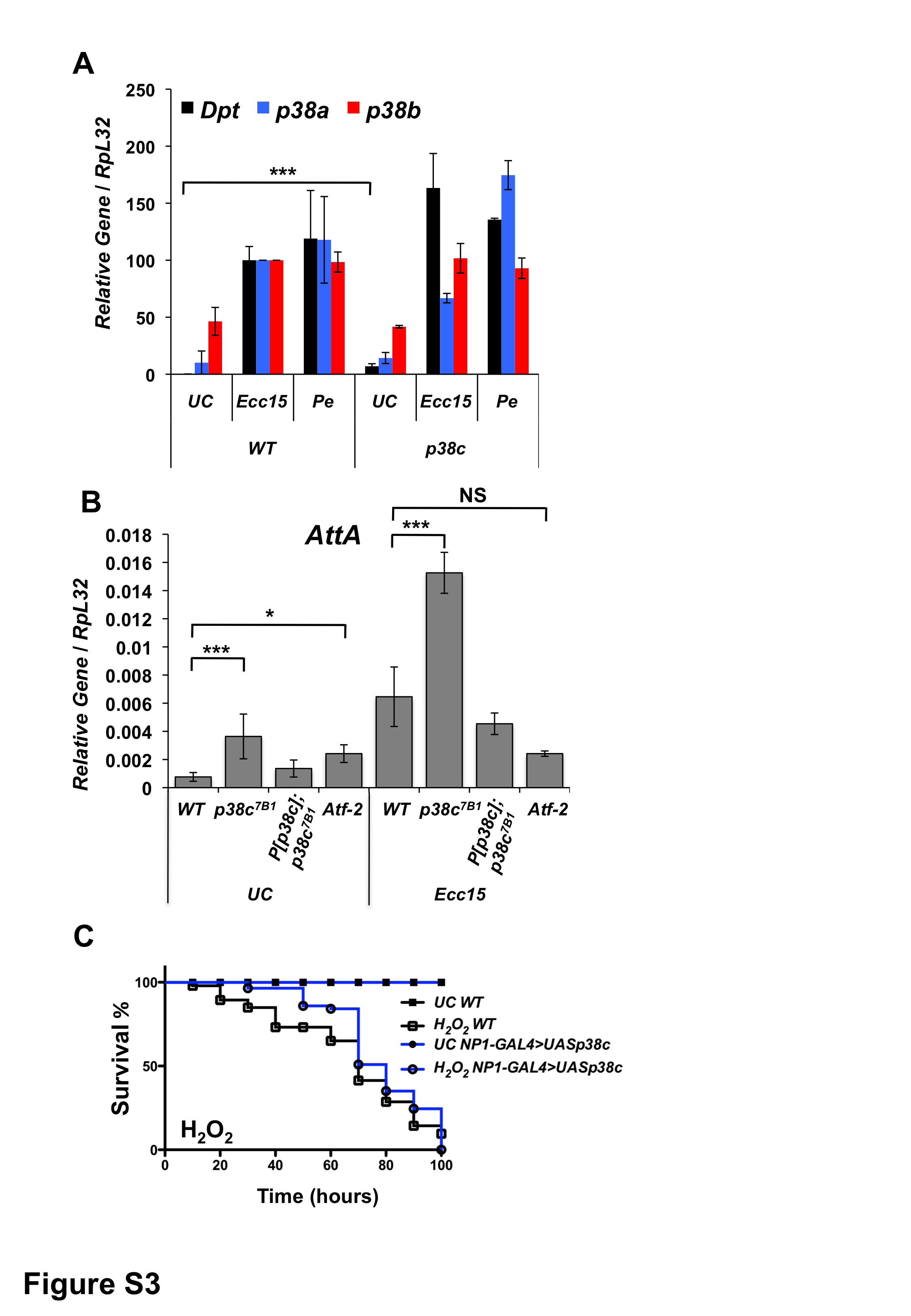

Supplement: Figure S3 — The expression of antimicrobial peptide genes is increased in the p38c mutant gut. (A) RT-qPCR analysis of Dpt, p38a and p38b expression in intestines of adult females either unchallenged or collected at 16h after oral infection with Ecc15 or P. entomophila. Dpt was up-regulated under basal conditions in the p38c7B1 mutant flies. *** p<0.001, determined by Student's t test. Data are the mean of three repeats and ± SE are shown. (B) Up-regulation of AttA expression in the p38c7B1 mutant was observed with or without infection. RT-qPCR was performed on total RNA extract from adult females intestine collected at 16 h after oral infection with Ecc15. UC: Unchallenged. NS: Not Significant (p = 0.3386); * p<0.05; *** p<0.001, determined by Student's t test. Data are the mean of three repeats and ± SE are shown. (C) Susceptibility to oxidative stress of wild-type flies (NP1-GAL4>Cs) and flies over-expressing p38c (NP1-GAL4>UAS-p38c) in the midgut fed on a diet with 1% H2O2. Despite an increase resistance at early time points, the survival of flies over-expressing p38c did not differ significantly from the wild-type based on a Kaplan-Meier log-rank. (TIF) [file pgen.1004659.s003.tif]

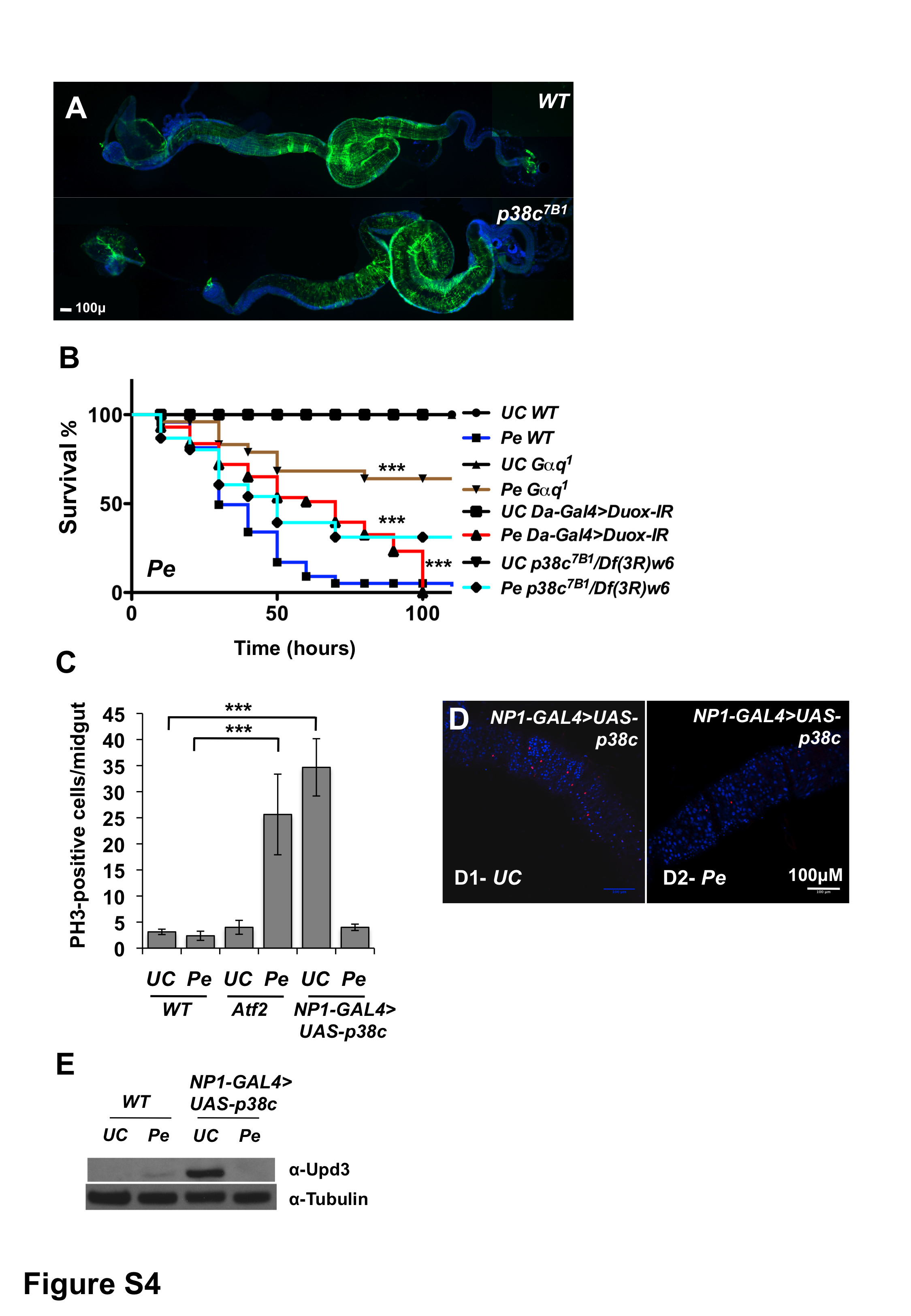

Supplement: Figure S4 — Contribution of p38c and Atf-2 to P. entomophila pathogenicity. (A) Structure and general organization of the gut of p38c deficient flies is similar to the wild-type. Green: visceral muscles stained with phalloidin-Alexa488; blue: nuclei marked with DAPI. (B) Gαq1 mutant, Duox RNAi and p38c/Df(3R)w6 flies exhibited an increased resistance to oral infection with P. entomophila. UC: unchallenged, Kaplan-Meier log-rank test used to determine statistical significance compared to the wild-type *** p<0.001. (C) P. entomophila infected atf-2 flies showed an increased mitotic index compared to wild-type flies. Flies over-expressing p38c (NP1-GAL4; UAS-p38c) had a higher mitotic index in absence of infection. Stem cell division along the midgut was quantified 8 h post-infection using an anti PH3-antibody. p<0.001 = *** (D) Immunostaining of guts revealed a higher number of mitotic stem cell in unchallenged flies over-expressing p38c (NP1-Gal4>UAS-p38c) D1. D2 flies were collected 8 h post-infection with P. entomophila. Mitotic stem cells: red; DAPI: blue. (E) Western blot analyses showed that flies over-expressing p38c have higher amount of Upd3 protein. Western blot was performed with protein extract of gut from flies either unchallenged or collected 16 h post-infection with P. entomophila. Flies that over-expressed p38c were subjected to P. entomophila mediated inhibition of translation and as consequence did not express Upd3 and did not show an increase of mitotic activity. (TIF) [file pgen.1004659.s004.tif]

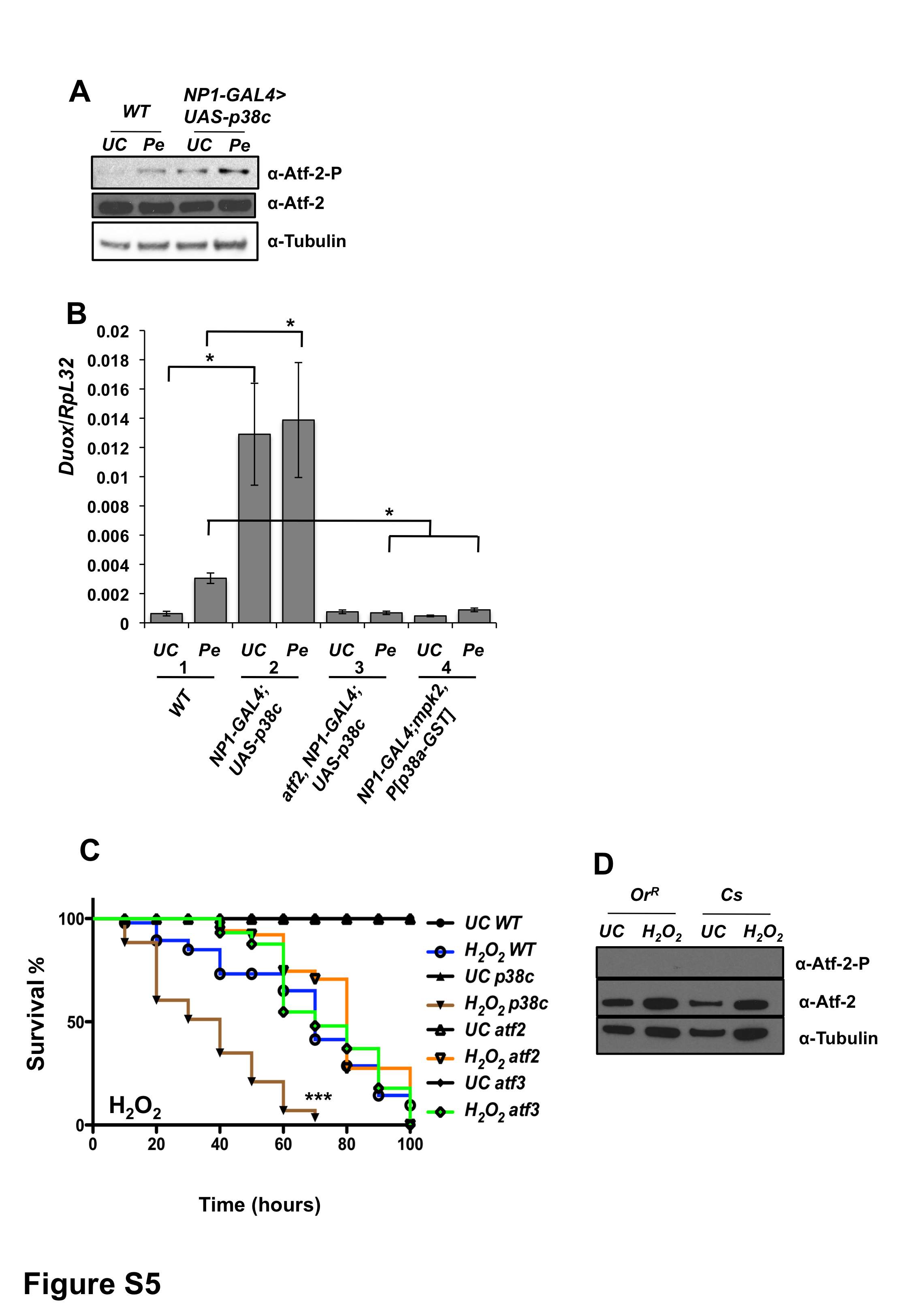

Supplement: Figure S5 — Atf-2 functions downstream of p38c in the regulation of Duox. (A) Western blot analysis showed an increase of Atf-2 phosphorylation where p38c was over-expressed. Guts were collected 4 h post-infection with P. entomophila. The total levels of Atf-2 remain unchanged in all genotypes with or without infection. (B) RT-qPCR analysis of Duox expression in various genetic backgrounds. Total RNA was extracted from guts of flies either unchallenged or collected 2 h after P. entomophila infection. Duox was highly expressed in absence of infection in flies over-expressing p38c but not in the Atf-2 mutant background. The induction of Duox upon P. entomophila infection was reduced in ‘p38a1’ mutant flies (deficient for both p38a and p38c) that over-express a functional p38a-GST fusion confirming that p38c is required for Duox up-regulation. The precise genotypes were 1. WT: NP1-GAL4/+, 2. NP1-Gal4/+;UAS-p38c/+, 3. Atf-2, NP1-Gal4/Atf-2, +; UAS-p38c/+ and 4. NP1-GAL4/+; p38a1, UAS-p38a-GST/p38a1,+. (C) Atf3 and Atf-2 mutant flies showed similar susceptibility to H2O2 as wild-type flies. A Kaplan-Meier log-rank test used to determine statistical significance. (D) Western blot analysis showed that Atf-2 phosphorylation was not induced when flies were fed on 1% H2O2. Flies were collected at 4 h post-feeding. (TIF) [file pgen.1004659.s005.tif]

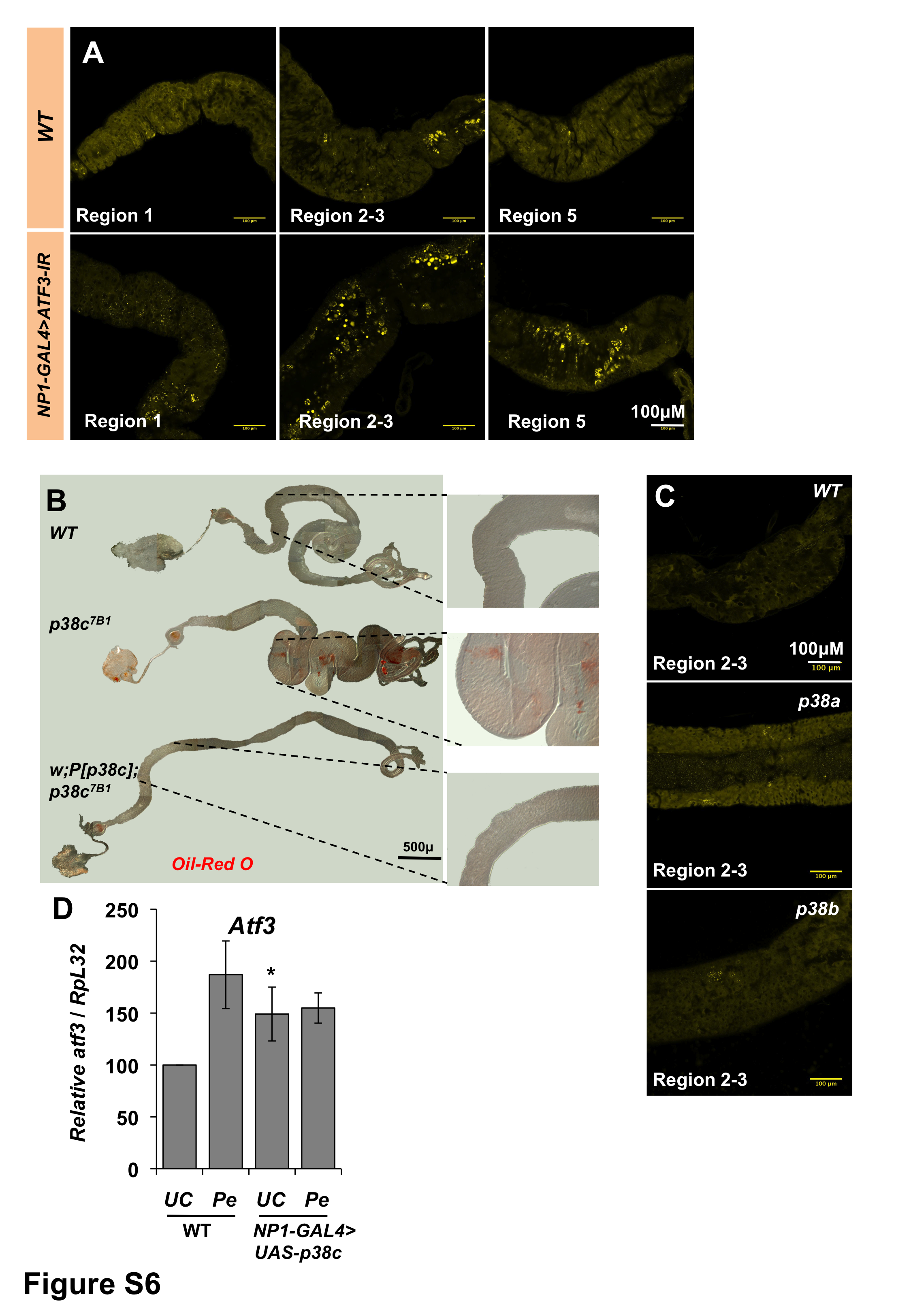

Supplement: Figure S6 — Increase accumulation of lipids in p38c7B1 fly intestines. (A) Silencing Atf3 by RNAi in the gut of adults leads to accumulation of lipids as observed by Nile Red staining. Different regions (Region 1, Regions2–3, Region 5) of the gut are shown for both the WT (NP1-GAL4/+ top panels) and Atf3 RNAi (NP1-GAL4; UAS-ATF3-IR bottom panels). (B) Oil-Red O stainings revealed a higher amount of lipids in the gut of p38c7B1 flies compared to the wild-type and P[p38c]; p38c7B1 flies. (C) p38a13 and p38b156A flies showed wild-type amounts of lipid in the intestine (WT: w1118). (D) The expression of atf3 increased in flies over-expressing p38c in the intestine WT: NP1-GAL4; + Data are the mean of three repeats and error bars show standard error. * p<0.05 as determined by Student's t test. (TIF) [file pgen.1004659.s006.tif]

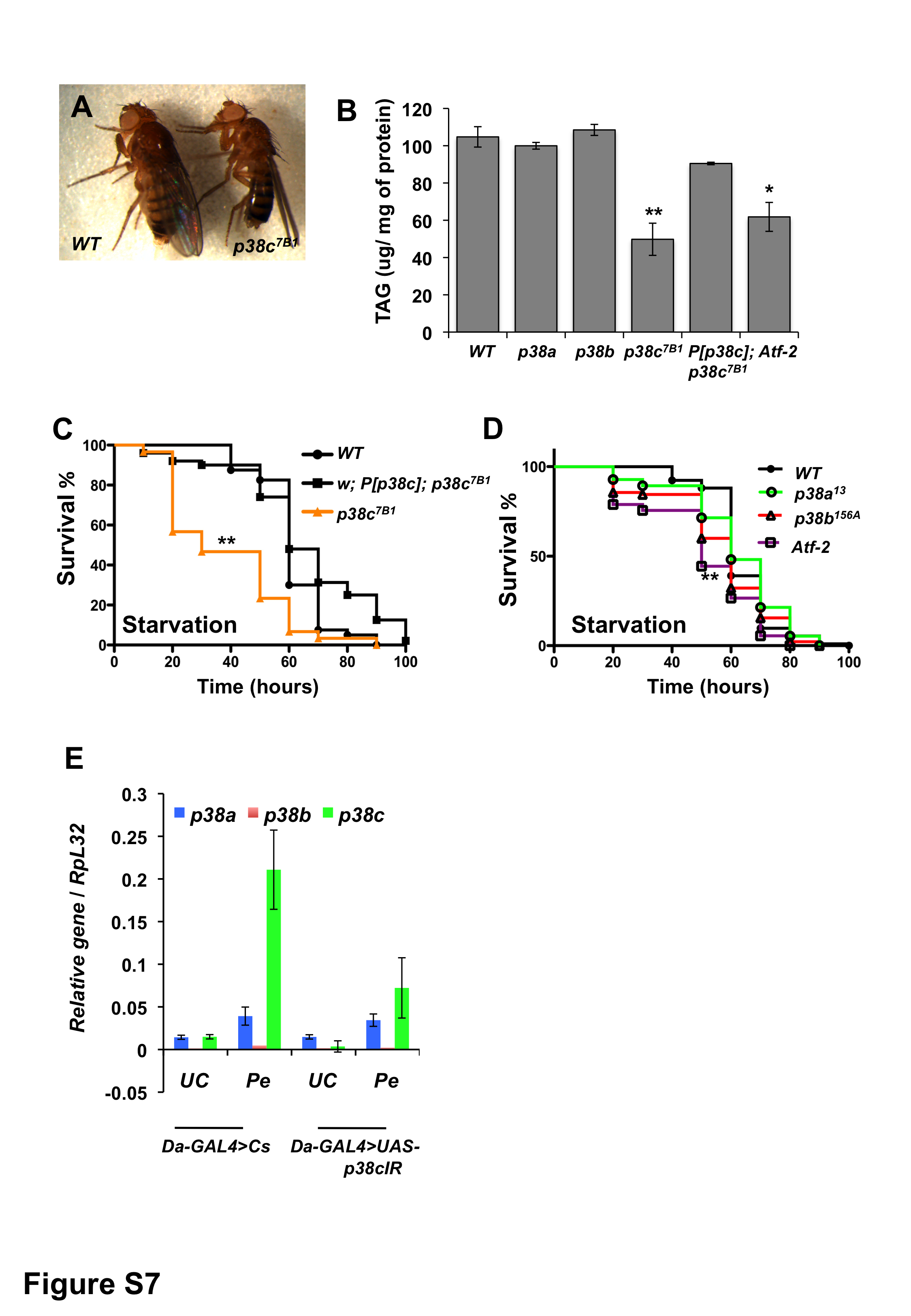

Supplement: Figure S7 — p38c flies have reduced TAG store. (A) p38c7B1 adult female flies appeared leaner (slightly smaller) than their wild-type (w1118) counterparts. Flies were imaged 3 days post-eclosion. (B) Shown are the levels of TAG in p38 and Atf-2 mutants relative to wild-type flies. Flies were maintained on standard Drosophila medium (see methods) for 3–5 days prior to TAG analysis. TAG measurements were normalized for the total amount of protein (µg/mg of protein). This analysis revealed that p38c and Atf-2 flies have lower levels of total TAG. Mean values of at least three experiments (N = decapitated 5 flies) (C & D) p38c mutant flies and to a lesser extent atf-2 flies exhibited an increase susceptibility to a starvation stress as compared to wild-type flies. 3–5 day-old females (genotype indicated in the panel) were fed on 1% agar vials. Kaplan-Meier log-rank test was used to determine statistical significance ** p<0.01. (E) Expression of a p38c-IR element with the ubiquitous driver Da-GAL4 reduced p38c expression without affecting p38a or p38b. RT-qPCR was performed on 3–5 days old flies. Genotype: Da-GAL4/+ and Da-GAL4/UAS-p38cIR. (TIF) [file pgen.1004659.s007.tif]

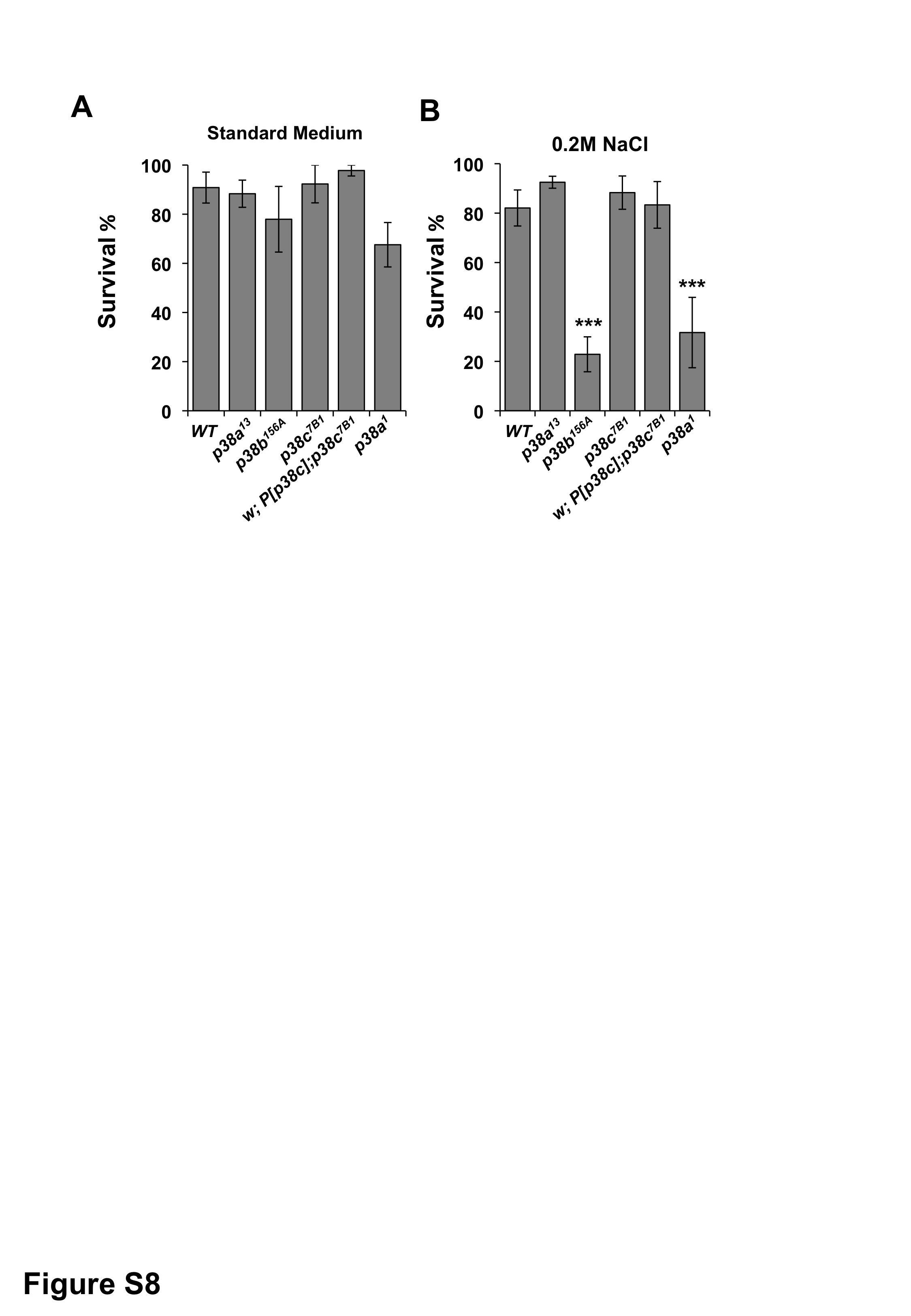

Supplement: Figure S8 — p38a, p38c double mutants are sensitive to salt stress. Embryos were placed on standard medium (A) or standard medium with 0.2 M NaCl (B), and the total numbers of offspring were counted. Panel B shows that p38b156A and mpk2 (deficient for both p38a and p38c) flies show an increase susceptibility to osmotic stress. WT: w1118 and other genotypes are indicated in the figure. (TIF) [file pgen.1004659.s008.tif]
