# Supplementary material for: The Drosophila MAPK p38c Regulates Oxidative Stress and Lipid Homeostasis in the Intestine
Source: PLoS Genet. 2014 Sep 25;10(9):e1004659. doi: 10.1371/journal.pgen.1004659 (PMC4177744; doi:10.1371/journal.pgen.1004659)
Supplement: Table S2 — List of qPCR primer sequences used in this study. (DOCX) [file pgen.1004659.s010.docx]

**Table S2. List of qPCR primer sequences used in this study**

| Gene | Left Primer 5’ to 3’ | Right Primer 5’ to 3’ |
| --- | --- | --- |
| *RpL32* | GACGCTTCAAGGGACAGTATCTG | AAACGCGGTTCTGCATGAG |
| *p38a* | CAGCCCGTGGGATCGGG | AGCTCCCGGTACGTCCTC |
| *p38b* | CGGCCAGGTCTGCAAGGC | CCATGTACACTTGCTGGAACTG |
| *p38c* | TACCTATCGCGAGATCCGTCT | ATGTACTTCAGTCCCCGCAGT |
| *LacZ* | CGAGGCGGTTTTCTCCGGCG | GCGTTAACTCGGCGTTTCATCTGTG |
| *Duox* | ACGTGTCCACCCAATCGCACGAG | AAGCGTGGTGGTCCAGTCAGTCG |
| *Atf3* | TGGTGGACATGCTGAAATCGC | ATGCTGCTGGTCAATCACGTT |
| *Dpt* | GCTGCGCAATCGCTTCTACT | TGGTGGAGTGGGCTTCATG |
| *AttA* | CCCGGAGTGAAGGATG | GTTGCTGTGCGTCAAG |
